# MiR-690, a Runx2-targeted miRNA, regulates osteogenic differentiation of C2C12 myogenic progenitor cells by targeting NF-kappaB p65

**DOI:** 10.1186/s13578-016-0073-y

**Published:** 2016-02-12

**Authors:** Shouhe Yu, Qianqian Geng, Qiuhui Pan, Zhongyu Liu, Shan Ding, Qi Xiang, Fenyong Sun, Can Wang, Yadong Huang, An Hong

**Affiliations:** Institute of Biomedicine, Jinan University, National Engineering Research Center of Genetic Medicine, Key Lab for Bioengineering Medicine of Guangdong Province, Guangzhou, Guangdong People’s Republic of China; Central Laboratory, People’s 10th Hospital, Shanghai, People’s Republic of China; College of Life Science, Yangtze University, Jingzhou, Hubei People’s Republic of China; Department of Materials Science and Engineering, Jinan University, Engineering Research Center of Artificial Organs and Materials, Ministry of Education, Guangzhou, Guangdong People’s Republic of China; Department of Medical Laboratory, People’s 10th Hospital, Shanghai, People’s Republic of China; College of Pharmacy, Jinan University, Guangzhou, Guangdong People’s Republic of China

**Keywords:** Runx2, miR-690, Osteogenic differentiation, p65, NF-κB pathway

## Abstract

**Background:**

The runt-related transcription factor 2 (Runx2) is a cell-fate-determining factor that controls osteoblast differentiation and bone formation. It has been previously demonstrated that microRNAs (miRNAs) play important roles in osteogenesis. However, the Runx2-regulated miRNAs that have been reported thus far are limited. In this study, we pursued to identify these miRNAs in Tet-on stable C2C12 cell line (C2C12/Runx2^Dox^ subline).

**Results:**

Microarray analysis revealed that alterations in miRNA expression occur with 54 miRNAs. Among these miRNAs, miR-690 was identified as a positive regulator of Runx2-induced osteogenic differentiation of C2C12 cells through gain- and loss-of-function assays. Expression of miR-690 is induced by Runx2, which binds directly to the putative promoter of *mir*-*690* (*Mirn690*). The miR-690 proceeds to inhibit translation of the messenger RNA encoding the nuclear factor kappa B (NF-κB) subunit p65 whose overexpression inhibits Runx2-induced osteogenic differentiation of C2C12 cells. Interleukin-6 (IL-6), a downstream target of NF-κB pathway, is upregulated by p65 overexpression but significantly downregulated during this differentiation process. Furthermore, overexpression of IL-6 impedes the expression of *osteocalcin*, a defined marker of late osteoblast differentiation.

**Conclusions:**

Together, our results suggest that the miR-690 transactivated by Runx2 acts as a positive regulator of Runx2-induced osteogenic differentiation by inactivating the NF-κB pathway via the downregulation of the subunit p65.

**Electronic supplementary material:**

The online version of this article (doi:10.1186/s13578-016-0073-y) contains supplementary material, which is available to authorized users.

## Background

The proper differentiation of mesenchymal progenitor cells into osteoblast lineage is pivotal for bone development in vivo. Osteoblast differentiation is governed by numerous regulatory pathways involving transcription factors, signaling molecules, and chromatin modifiers [1–3]. In addition, posttranscriptional mechanisms including miRNA-mediated regulation also play important roles in regulating osteogenic differentiation.

MicroRNAs (miRNAs) are small (approximately 22nt), functional, highly conserved, endogenous noncoding RNA. They regulate protein translation or mRNA stability by imperfect binding to the 3′ untranslated region (3′UTR) of their target genes. These small RNAs have emerged as key regulators of almost every biological process in eukaryotes, including early development, growth and differentiation, and cell apoptosis. The impact of miRNAs on osteoblast differentiation has been investigated in several studies. It has been shown that miR-135 targets the intracellular receptor *SMAD family member 5* and inhibits bone morphogenetic protein 2(BMP-2)-induced osteogenic differentiation of C2C12 cells [4]. MiR-141 and its homolog miR-200a are involved in BMP-2-induced preosteoblast differentiation by regulating their common target *distal*-*less homeobox 5* [5]. In primary mouse osteoblasts, miR-93 has been found to target *osterix* and then inhibits osteoblast mineralization [6]. MiR-204 and its homolog miR-211 stimulate adipocyte differentiation but inhibit osteoblast differentiation of bone marrow stromal cells through targeting *runt*-*related transcription factor 2* (*Runx2*) [7]. In addition, a panel of 11 miRNAs, including miR-204, has been found to control osteogenic lineage progression by targeting *Runx2* [8]. These results suggest that the expression level of osteogenesis-related factors could be tightly controlled by miRNAs, which is an effective method for regulating osteogenesis. Additionally, the miRNAs controlled by these factors (especially the osteoblast-specific transcription factors) are also indispensable for regulating osteogenesis.

Runx2 (also known as Cbfa1, AML-3, and PEBP2aA), an osteoblast-specific transcription factor belonging to the runt-domain gene family [9], is expressed in mesenchymal condensations during early embryonic development [10]. Many previous studies have revealed that during both embryogenesis and postnatal life, Runx2 plays a key role in regulating osteoblast and hypertrophic chondrocyte differentiation, and bone formation [11–13]. The underlying mechanism by which Runx2 regulates these processes is still unclear, and the identification of Runx2-regulated miRNAs can provide new insight into the mechanism. However, Runx2-controlled miRNAs that have been identified thus far are limited.

In the current study, we explored Runx2-regulated miRNAs by using C2C12/Runx2^Dox^ subline previously described [14], and obtained 54 differentially expressed miRNAs during Runx2-induced osteogenic differentiation, and identified miR-690 as a positive regulator during this differentiation process. Furthermore, we demonstrated that miR-690 is directly induced by Runx2. The nuclear factor kappa B (NF-κB) subunit p65 was identified as a direct target of miR-690, and its overexpression inhibits Runx2-induced osteogenic differentiation. Interleukin-6 (IL-6), a downstream target of NF-κB pathway [15], is upregulated by p65 overexpression but significantly downregulated during this differentiation process. Furthermore, overexpression of IL-6 impedes Runx2-induced late osteoblast differentiation. Together, our findings suggest that Runx2 promotes osteogenic differentiation of C2C12 cells by inactivating NF-κB pathway via the upregulation of miR-690.

## Results

### Identification of differentially expressed miRNAs during Runx2-induced osteogenic differentiation

Elucidating Runx2-regulated miRNAs could provide new insight into the mechanism whereby Runx2 functions in osteogenesis. Here, we used the C2C12/Runx2^Dox^ subline previously described [14] to explore this question. In C2C12/Runx2^Dox^ cells, the expression of Flag-Runx2 can be tightly regulated by doxycycline (Dox) treatment. In the absence of Dox, the C2C12/Runx2^Dox^ cells cultured in medium containing 2 % horse serum can be induced into multinucleated myotubes (Additional file 1: Figure S1A). Additionally, C2C12/Runx2^Dox^ cells also can be induced into osteoblasts by BMP-2 treatment in the absence of Dox (Additional file 1: Figure S1B). These results suggest that the differentiation characteristics of C2C12/Runx2^Dox^ cells is similar to that of C2C12 myogenic progenitor cells. Taken together, we believed that the C2C12/Runx2^Dox^ cells are actually the wild type C2C12 myogenic progenitor cells in the absence of Dox.

**Fig. 1:**
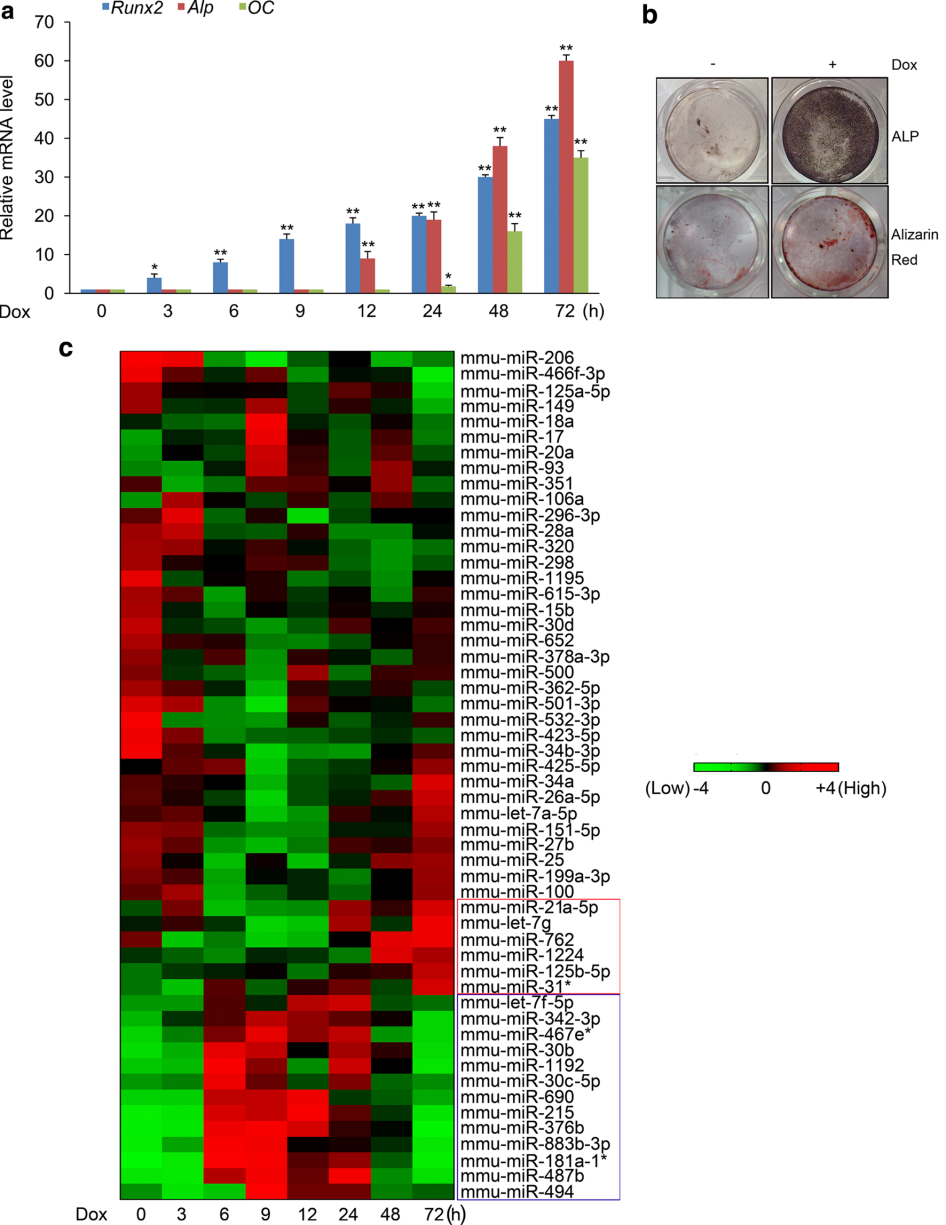
A program of miRNAs is expressed during Runx2-induced osteogenic differentiation of C2C12 cells. **a** C2C12/Runx2^Dox^ cells were treated with Dox for the times indicated (0–72 h). The mRNA level of *Runx2*, *Alp*, and *OC* was determined by real-time qPCR. All data are presented as the mean ± SD from three independent experiments. *P < 0.05, **P < 0.01 compared with untreated cells (0 h). **b** ALP and matrix mineralizing activity were measured by ALP and Alizarin red staining at days 7 and 28, respectively. Similar results were obtained in three independent experiments. **c** Differentially expressed miRNAs from miRNA microarray data. 54 miRNAs whose expression changed by ≥ 1.5-fold at least one time point were chosen as the differentially expressed miRNAs by comparing with untreated cells (0 h), and these miRNAs were arranged by unsupervised hierarchical clustering. Data value displayed as *red* and *green* represent elevated (high) and reduced expression (low), respectively

Osteogenic differentiation of C2C12 cells was evidenced by increased expression of genes associated with osteoblast differentiation, *alkaline phosphatase* (*Alp*) and *osteocalcin* (*OC*), at the indicated times after Dox treatment (Fig. 1a). The expression of mRNA and protein for Flag-Runx2 was increased in a time-dependent manner (Fig. 1a, Additional file 2: Figure S2A). In immunocytochemistry analysis, Flag-Runx2 was found to be mainly expressed in the nuclei of Dox-treated C2C12/Runx2^Dox^ cells, and the percentage of cells overexpressing Flag-Runx2 was also increased in a time-dependent manner (Additional file 3: Figure S3). The osteoblast phenotype was confirmed by demonstration of increased ALP activity and Alizarin Red staining for matrix mineralization (Fig. 1b). These results suggest that the C2C12/Runx2^Dox^ subline works well. Moreover, the cell line harboring the empty vector (C2C12-empty vector Tet-on cell line, C2C12/vector^Dox^ cells) was used to investigate and exclude the influence of Dox. As shown in Additional file 4: Figure S4, the ALP activity and matrix mineralization were absent in Dox-treated C2C12/vector^Dox^ cells. These results suggest that Dox itself has no effect on the osteogenic differentiation of C2C12 cells.

To detect miRNAs related to Runx2-induced osteogenic differentiation, miRNA profiling was performed by using total RNA from Dox-treated C2C12/Runx2^Dox^ cells collected at 0, 3, 6, 9, 12, 24, 48, and 72 h. The values of the miRNA expression level at each time point were clustered and graphically illustrated (Additional file 5: Figure S5). Expression levels of 54 miRNAs were altered during this differentiation process (Fig. 1c). Of these, 13 miRNAs were upregulated in the early induction phase (6–24 h), and declined to basal levels in the late induction phase (48 and 72 h) (blue box in Fig. 1c). As shown in Fig. 1a and Additional file 2: Figure S2, C2C12/Runx2^Dox^ cells were induced into osteoblasts by 12 h based on expression of muscle and bone phenotypic genes, suggesting the crucial role of early induction phase in Runx2-induced transdifferentiation of myoblasts (C2C12 cells) into the osteoblastic phenotype. Thus, for osteogenesis to proceed, there is a requirement for upregulation of the 13 miRNAs in the dataset responsive to Runx2 overexpression to convert the differentiation pathway of C2C12 myoblasts into the osteoblast lineage. We then focused our attention on these miRNAs.

### Effect of miR-690 on Runx2-induced osteogenic differentiation

**Fig. 2:**
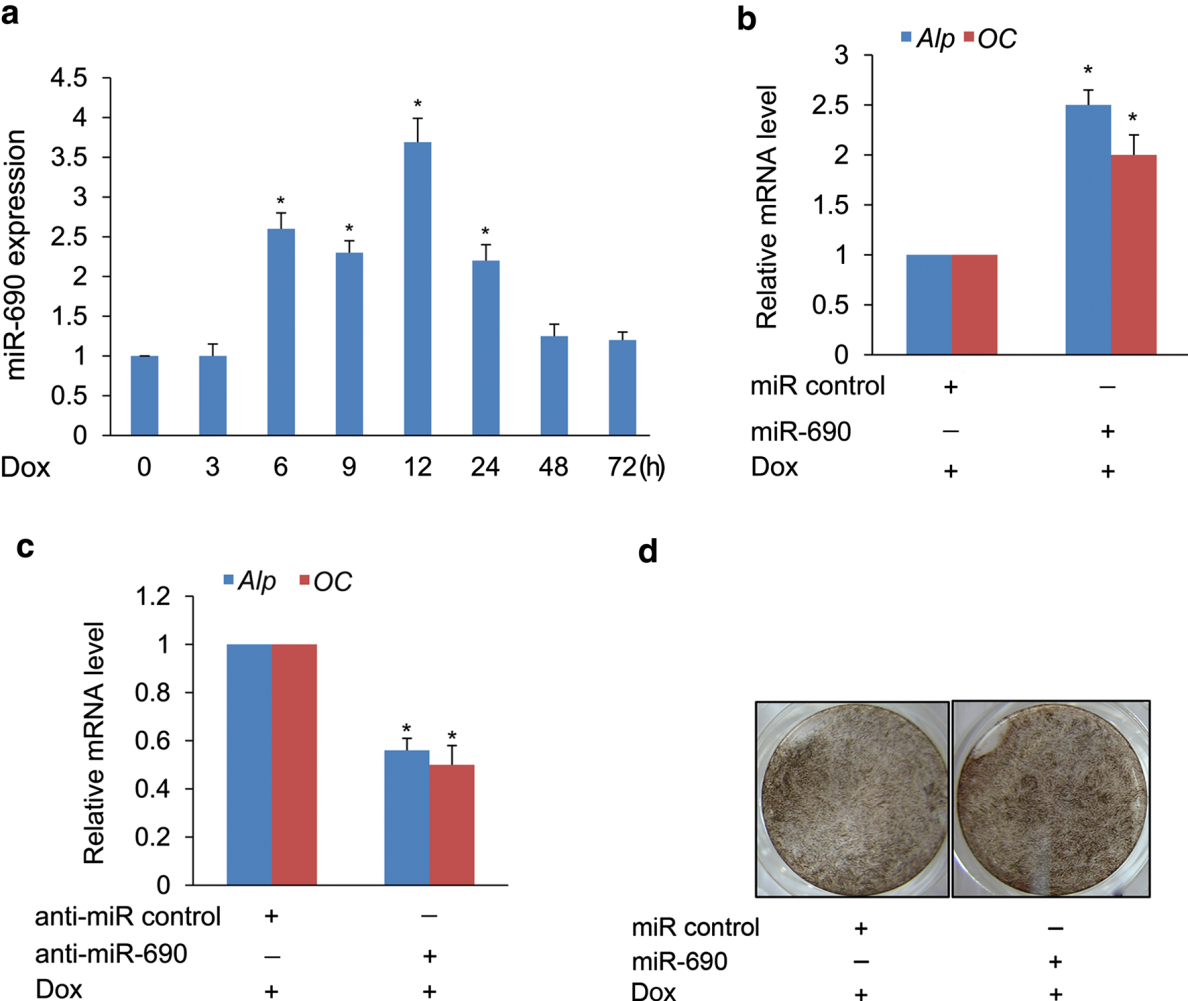
miR-690 synergistically potentiates Runx2-induced osteogenic differentiation. **a** Real-time qPCR analysis of miR-690 was performed in Dox-treated samples used for miRNA profiling analysis. **b**, **c** Effect of miR-690 on expression of osteoblastic markers. C2C12/Runx2^Dox^ cells were transfected with miR control (**b**), miR-690 (**b**), anti-miR control (**c**), and anti-miR-690 (**c**), respectively, and then treated with Dox for 3 days. The mRNA level of *Alp* and *OC* was measured by real-time qPCR. *P < 0.05 compared with miR control or anti-miR control samples. **d** Effect of miR-690 on ALP activity. C2C12/Runx2^Dox^ cells were transfected with miR control and miR-690, respectively, and then treated with Dox for 7 days. The ALP activity was determined by ALP staining. Similar results were obtained in three independent experiments

To evaluate the biological effect of the above-mentioned 13 miRNAs, anti-miRs and miRNA mimics for these miRNAs were used in a functional screening for their impact on Runx2-induced osteogenic differentiation. Here, we identified miR-690 as a potential positive regulator of Runx2-induced osteogenic differentiation. Expression level of miR-690 was measured at different time points during this differentiation process. MiR-690 was significantly upregulated at hour 6, and remained upregulated up to hour 24, and declined to basal levels at hour 48 and 72 respectively (Fig. 2a). Additionally, Dox itself also has no effect on the expression of miR-690 (Additional file 6: Figure S6). The miR-690 expression change assayed by real-time quantitative PCR (qPCR) was generally similar to the change in miR-690 expression as determined by miRNA microarray (Fig. 1c).

To study the effect of miR-690 on Runx2-induced osteogenic differentiation, we transfected miR control, miR-690, anti-miR control, and anti-miR-690 into C2C12/Runx2^Dox^ cells, respectively, and found that transfection of miR-690 increased expression of *Alp* and *OC* (Fig. 2b), whereas transfection of anti-miR-690 reduced their expression (Fig. 2c). Moreover, ALP activity was slightly increased in miR-690-transfected C2C12/Runx2^Dox^ cells (Fig. 2d). These findings demonstrate that miR-690 synergistically potentiates Runx2-induced osteogenic differentiation of C2C12 cells.

### *Mir*-*690* is a direct transcriptional target of Runx2

**Fig. 3:**
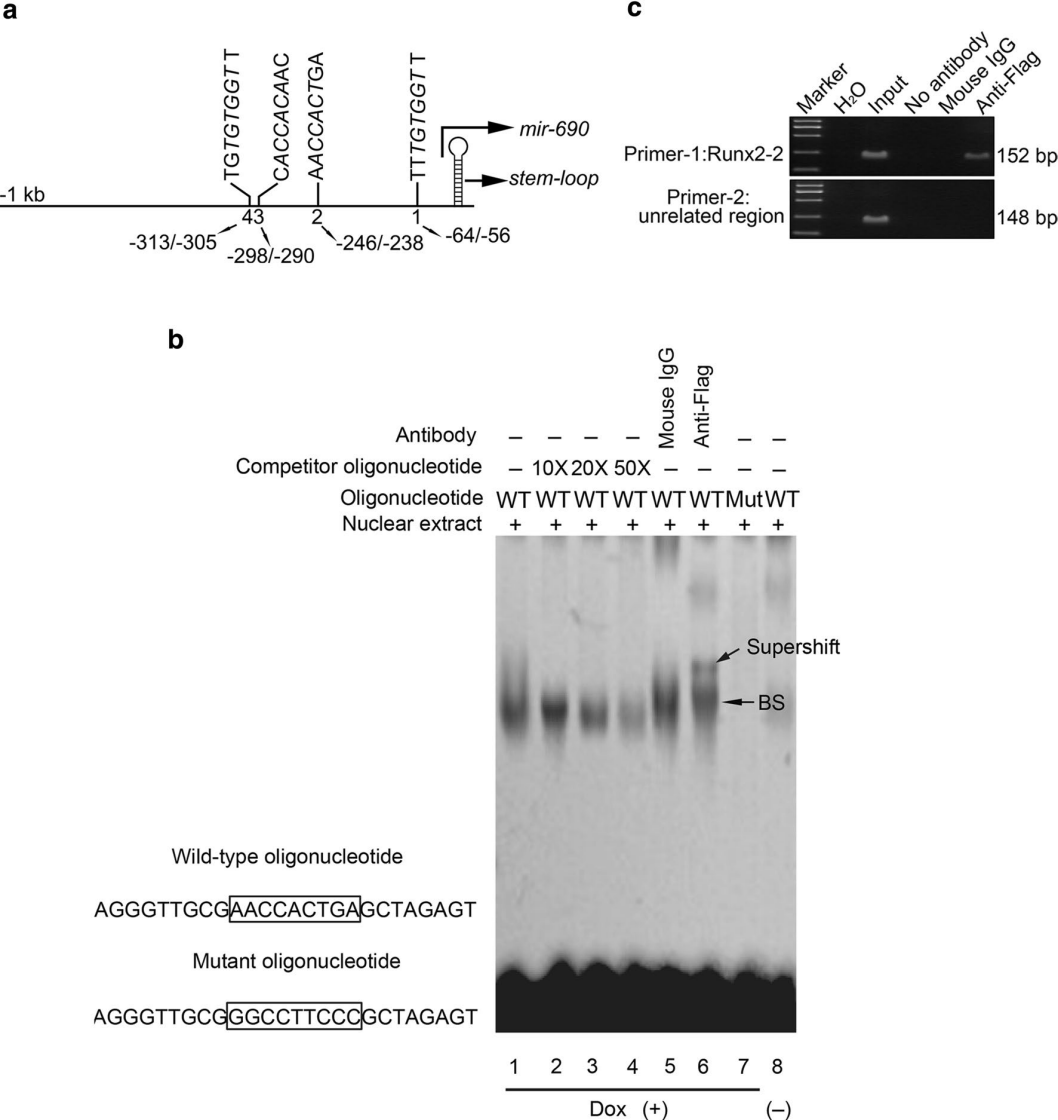
miR-690 is directly regulated by Runx2. **a** Schematic representation of mouse *mir*-*690* genomic locus, which indicates the locations of the four potential Runx2-binding sites. The *italic letters* indicate the core sequence of each site. **b** EMSA shows the interaction between *mir*-*690* site (Runx2-2) and Runx2 in Dox-treated cells. The *bottom arrow* indicates the DNA–protein complex (BS). The *top arrow* indicates the Runx2-supershifed complex. *WT* wild-type, *Mut* mutant. **c** ChIP analysis was performed to confirm the interaction of Runx2 with *mir*-*690* promoter in vivo. PCR was performed with primer-1, which was designed to amplify a fragment of the *mir*-*690* promoter flanking the Runx2-2 site. The primer-2 for an unrelated part (−1800/− 1653) in the distal 5′ flanking region of the Runx2-2 site was utilized for the control reaction in this ChIP analysis

*Mir*-*690* expression can be rapidly induced following activation of Runx2 (Fig. 2a), raising the possibility that *mir*-*690* is a direct target of Runx2. To test this hypothesis, we first examined the −1 kb genomic sequence upstream of the *mir*-*690* stem-loop to search for potential Runx2-binding site using TFSEARCH database [16] and ConSite program [17]. Indeed, we identified four potential sites at −313 to −305 (site 4, Runx2-4), −298 to −290 (site 3, Runx2-3), −246 to −238 (site 2, Runx2-2), and −64 to −56 (site 1, Runx2-1), respectively (Fig. 3a). With an electrophoretic mobility shift assay (EMSA), the labeled oligonucleotide containing Runx2-2 produced a significant shift (BS) in the Dox-treated cells nuclear extract (Fig. 3b, BS in lane 1), but the labeled oligonucleotides containing Runx2-1, Runx2-3, or Runx2-4 failed to do so (data not shown). No apparent shift was produced by the oligonucleotide containing Runx2-2 in the extract from non-treated cells (Fig. 3b, lane 8). The observed shift was abolished by 10- to 50-fold molar excess of unlabeled oligonucleotides (Fig. 3b, lanes 2–4). The labeled oligonucleotide contained a 9 bp mutation also abolished the shift (Fig. 3b, lane 7). Furthermore, the anti-Flag antibody, not the mouse IgG antibody, supershifted the complex (BS) (Fig. 3b, lanes 5 and 6), suggesting the specificity of the interaction. Thus, the EMSA assay demonstrated that the *mir*-*690* promoter contains one Runx2-binding site, Runx2-2.

Chromatin immunoprecipitation (ChIP) assay was performed to examine whether Runx2 interacts with *mir*-*690* promoter in vivo. As shown in Fig. 3c, the anti-Flag antibody specifically enriched the regions containing Runx2-2 (primer-1). By contrast, no positive signals were detected in various negative controls, including the PCR control (H_2_O) or immunoprecipitation control. Additionally, Runx2 did not associate with the unrelated part in the distal 5′ flanking region of the Runx2-2 site (primer-2). These results indicate that Runx2 binds to the *mir*-*690* promoter in vivo. Together, these results suggest that Runx2 acts as a transcriptional activator on *mir*-*690*.

### MiR-690 directly targets *p65* through translational inhibition

**Fig. 4:**
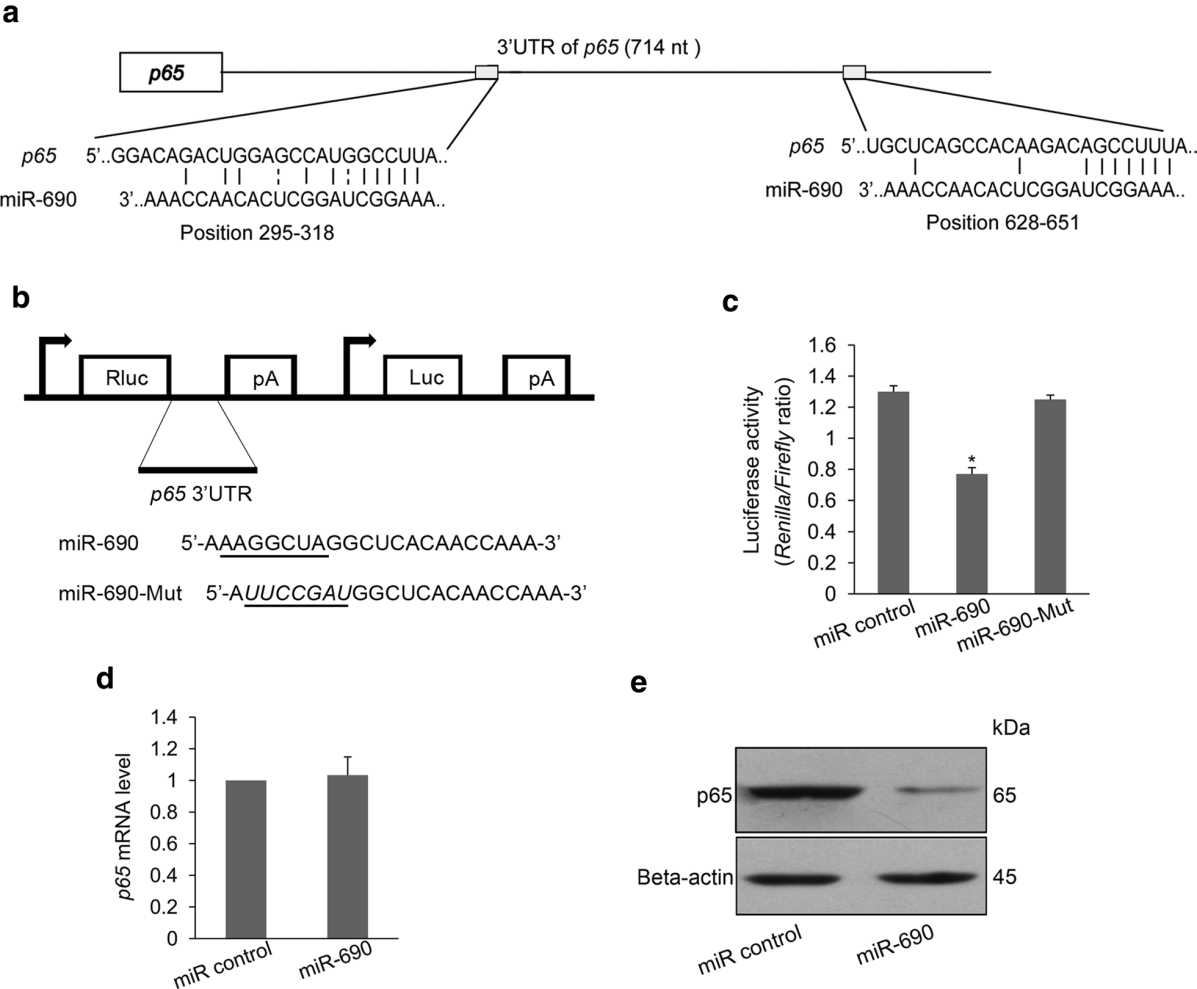
miR-690 directly targets *p65*. **a** Schematic representing miR-690 target sites in *p65* 3′UTR and base-pairing of miR-690 sequences with the 3′UTR. **b** Schematic diagram illustrating the design of luciferase reporter with *p65* 3′UTR. The sequences of miR-690 and miR-690-Mut are also shown. The *underlined* sequences represent wild-type and mutated seed sequences of miR-690. *Rluc* Renilla luciferase, *Luc* Firefly luciferase, *pA* polyadenylation signal. **c** miR control, miR-690, or miR-690-mut was co-transfected with psiCHECK-2-*p65* 3′UTR reporter into C2C12/Runx2^Dox^ cells. After 48 h, cells were harvested and luciferase activities were measured. Luciferase activity = (*Renilla*/*Firefly*) ratio. Renilla, raw renilla luciferase activity; Firefly, firefly luciferase activity. Data are presented as mean ± SD (n = 3). *P < 0.05 compared with miR control sample. **d**, **e** Relative mRNA level of *p65* (**d**) and protein level of p65 (**e**) in miR control- or miR-690-transfected cells

To understand the molecular mechanism that underlies miR-690-mediated regulation, we search for potential targets of miR-690 implicated in Runx2-induced osteogenic differentiation using the miRNA target prediction algorithms DIANA-microT, miRDB, and miRWalk [18–20]. More than 100 genes were predicted to be potential target genes for miR-690. Among the predicted genes, we identified p65, a NF-κB subunit with a negative role in osteoblast differentiation [21]. According to in silicon analysis, *p65* has two potential binding sites for miR-690 within its 3′UTR (Fig. 4a). To investigate whether *p65* can be directly targeted by miR-690, we engineered a luciferase reporter that has wild-type 3′UTR of *p65* (Fig. 4b). Co-transfection of the *p65* 3′UTR luciferase reporter with miR-690 resulted in downregulation of the luciferase activity compared with the miR control (Fig. 4c). In comparison, miR-690-Mut had no effect on *p65* 3′UTR luciferase reporter (Fig. 4c), implying that *p65* is a direct target of miR-690. Thus, we further examined how miR-690 repressed p65 expression. Real-time qPCR results revealed no significant change in *p65* mRNA level when miR-690 was transfected into C2C12/Runx2^Dox^ cells (Fig. 4d). However, Western blot result showed reduced p65 protein level in the miR-690-transfected cells compared with miR control sample (Fig. 4e), indicating that miR-690 mediates translational repression but not mRNA degradation of *p65*. Additionally, the expression of p65 was slightly increased at the protein but not the mRNA level in the anti-miR-690-transfected cells compared with anti-miR control sample (Additional file 7: Figure S7). Together, these results indicate that miR-690 directly targets *p65* through translational inhibition.

### p65 exerts its effect on Runx2-induced osteogenic differentiation partially through IL-6 upregulation

**Fig. 5:**
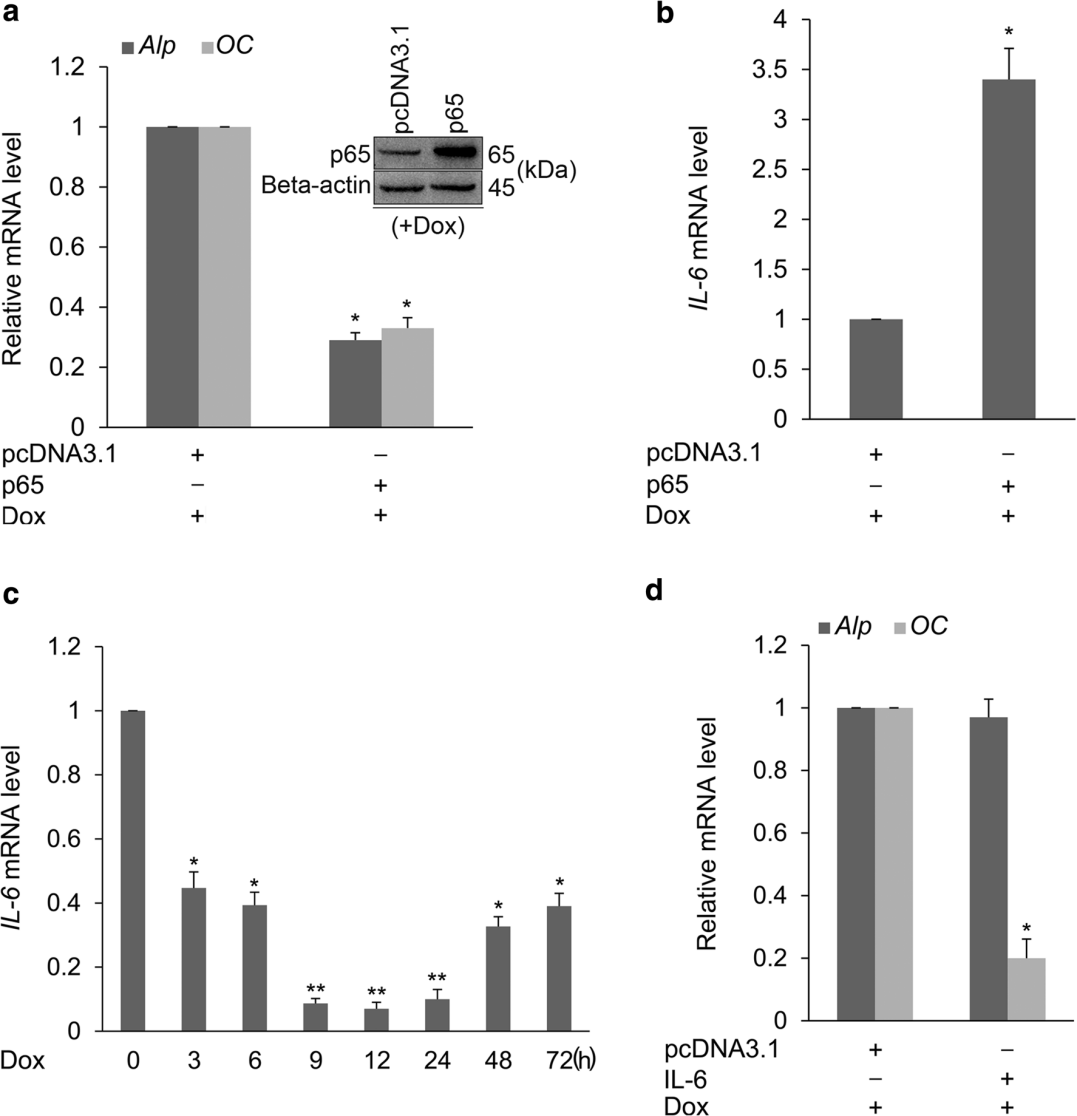
p65 exerts its effect on Runx2-induced osteogenic differentiation partially through IL-6 upregulation. **a**, **b** The empty vector (pcDNA3.1) or p65 expression vector (pcDNA3.1-*p65*) was transfected into C2C12/Runx2^Dox^ cells. The transfected cells were treated with Dox for 3 days and subjected to real-time qPCR analysis for *Alp* (**a**), *OC* (**a**) and *IL*-*6* (**b**) mRNAs, respectively. Data are presented as mean ± SD (n = 3). *P < 0.05 compared with pcDNA3.1-transfected cells. Western blot was performed to assess the overexpression level of p65 (**a**, *inset*). **c** Real-time qPCR results of *IL*-*6* in Dox-treated cells. Data are presented as mean ± SD (n = 3). *P < 0.05, **P < 0.01 compared with untreated cells (0 h). **d** The empty vector (pcDNA3.1) or IL-6 expression vector (pcDNA3.1-*IL*-*6*) was transfected into C2C12/Runx2^Dox^ cells. The transfected cells were treated with Dox for 3 days and subjected to real-time qPCR analysis for *Alp* and *OC* mRNAs. Data are presented as mean ± SD (n = 3). *P < 0.05 compared with pcDNA3.1-transfected cells

Since *p65* is directly targeted by miR-690, it was necessary to examine whether p65 exerts an opposite effect on Runx2-induced osteogenic differentiation as compared with miR-690. *p65* expression vector was used for transfection of C2C12/Runx2^Dox^ cells, followed by Dox treatment for 3 days. Real-time qPCR data revealed that *p65* overexpression attenuates the expression of *Alp* and *OC* (Fig. 5a), suggesting that p65 is a negative regulator of Runx2-induced osteogenic differentiation. Additionally, the mRNA level of p65 was unchanged during this differentiation process (Additional file 8: Figure S8A). However, the protein level of p65 was decreased in Dox-treated C2C12/Runx2^Dox^ cells at hour 12, possibly resulting from the high level of miR-690 at this time point (Additional file 8: Figure S8B). This finding shows that *p65* is indeed a direct target of miR-690 during this differentiation process.

Previous studies have shown that *IL*-*6* is a direct transcriptional target of NF-κB [15], and that osteoblast differentiation can be negatively regulated by IL-6 [22]. This observation raised the possibility that p65 may exert its negative effect on Runx2-induced osteogenic differentiation by upregulating IL-6. We therefore performed a series of experiments to investigate this possibility. *IL*-*6* expression was found to be upregulated in C2C12/Runx2^Dox^ cells transfected with p65 expression vector in the presence of Dox for 3 days (Fig. 5b). In the absence of Dox, *IL*-*6* expression was also upregulated by p65 overexpression (Additional file 9: Figure S9). These results suggest that *IL*-*6* could be activated by p65 during this differentiation process. To further verify the above-mentioned possibility, we also examined the mRNA level of *IL*-*6* in Dox-treated C2C12/Runx2^Dox^ cells, and found that the expression of *IL*-*6* was significantly downregulated (Fig. 5c), implying the negative effect of IL-6 on Runx2-induced osteogenic differentiation. In addition, the role of IL-6 in the differentiation process of C2C12/Runx2^Dox^ cells was examined by transfection of *IL*-*6* expression vector. As shown in Fig. 5d, the expression of *OC*, a defined marker of late osteoblast differentiation, was significantly downregulated by IL-6 overexpression. However, the expression of *Alp*, a marker of early osteoblast differentiation, was not affected. Additionally, the negative effect of p65 on the expression of *OC* was attenuated in si-IL-6-transfected C2C12/Runx2^Dox^ cells compared with si-NC sample (Additional file 10: Figure S10). Taken together, these results indicate that p65 exerts its negative effect on Runx2-induced osteogenic differentiation partially through IL-6 upregulation.

## Discussion

**Fig. 6:**
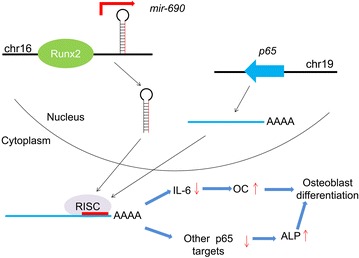
Proposed model for the regulation and function of miR-690 during Runx2-induced osteogenic differentiation. *Chr* chromosome, *RISC* RNA-induced silencing complex

In the present study, we reported an extensive genomic-wide profiling of miRNA expression during Runx2-induced osteogenic differentiation, and identified 54 differentially expressed miRNAs. MiR-690, a newly identified Runx2-targeted miRNA, was found to promote Runx2-induced osteogenic differentiation through translational inhibition of its target *p65*, permitting the downregulation of the expression of p65 downstream targets (IL-6 and other undisclosed targets); this favours, in turn, Runx2-induced osteogenic differentiation (Fig. 6).

Balint et al. have demonstrated that induction of osteogenic differentiation of C2C12 cells by BMP-2 could be separated into several distinct stages, and that the 8–12 h window may make the point of osteoblast phenotypic determination, and that the bone phenotype can be further established from 16 to 24 h [23]. These results are consistent with the finding reported by Li et al. [4]. In our study, the commitment of C2C12 myoblasts to the osteogenic phenotype is recognized by 12 h but not by 8 h (Fig. 1a). The discrepancy between BMP-2- and Runx2-induced systems might arise from the different osteogenic effect of BMP-2 and Runx2 on C2C12 cells. Taken together, we concluded that the period from 12 to 24 h appears to be a point of osteoblast phenotypic determination, and that the period from 24 to 72 h might be used for further establishing bone phenotype during Runx2-induced osteogenesis.

Many researches have shown that miRNAs play an important role in regulating osteoblast differentiation and bone formation [24]. A panel of miRNAs has been reported to control osteogenesis by targeting the cell-fate-determining transcription factor Runx2 [8], but the Runx2-regulated miRNAs that have been identified are still limited (e.g., miR-2861-3960 cluster) [25]. In this study, we performed a miRNA microarray by using total RNA from Dox-treated C2C12/Runx2^Dox^ cells, and identified 54 differentially expressed miRNAs regulated by Runx2. During Runx2-induced osteogenic differentiation of C2C12 cells, the microarray data could reveal the followings: First, 19 miRNAs were upregulated and 35 miRNAs were downregulated during osteogenic differentiation from 609 mouse miRNAs on the array. Second, the 35 downregulated miRNAs were predicted to target mainly regulatory factors that function to promote myogenesis and inhibit osteogenesis independently. MiR-206, a significantly downregulated miRNA in microarray data (Fig. 1c), was reported to be a muscle-specific miRNA and to promote myoblast differentiation by directly targeting *paired box 7* [26, 27]. MiR-466f-3p and miR-125a-5p, whose expression were also downregulated (Fig. 1c), were found to be significantly downregulated during osteogenic differentiation of bone marrow- and adipose tissue-derived mesenchymal stem cells [28, 29], suggesting the general role of these miRNAs in the inhibition of osteoblast differentiation. However, the bona fide targets of these miRNAs are still unclear during osteogenesis. Third, the 19 upregulated miRNAs could be divided into two categories: 13 miRNAs whose expression was upregulated in the early induction phase (6-24 h) but declined to basal levels in the late induction phase (48 and 72 h) (blue box in Fig. 1c), and 6 miRNAs whose expression was only significantly upregulated in the late induction phase (48 and 72 h) (red box in Fig. 1c). The distinct expression pattern of 19 upregulated miRNAs revealed that these miRNAs might exert different effect on Runx2-induced osteogenic differentiation. MiR-21a-5p, miR-762, and miR-1224, belonging to the 6 upregulated miRNAs, were reported to be involved in bone matrix mineralization [30–32], suggesting that the 6 upregulated miRNAs might participate in the regulation of Runx2-induced late osteoblast differentiation.

Here, we concluded that the 13 upregulated miRNAs might play a key role in the commitment of C2C12 cells into osteoblastic lineage. To evaluate the biological effect of the 13 upregulated miRNAs on Runx2-induced osteogenic differentiation, anti-miRs and miRNA mimics for these miRNAs were used in a functional screening. Finally, the screening identified miR-690 as a candidate regulator of Runx2-induced osteogenic differentiaition. Previous study has reported that miR-690 might play an important role in glucose regulation of β-cell function [33]. MiR-690 is highly overexpressed in functional myeloid-derived suppressor cells (MDSCs) and manipulates MDSC activity by repressing transcription factor CCAAT/enhancer-binding protein α in inflammatory diseases as well as cancer [34, 35]. MiR-690 can also regulate fibroblast migration and dermal wound repair by directly targeting *Versican* [36]. In addition, miR-690 was found to be involved in granulopoiesis [37], glutamate-induced excitotoxicity [38], and regulating testosterone signaling in liver [39]. However, the function of miR-690 in regulating osteogenic differentiation was not clear. In this study, data obtained from in vitro experiments revealed that miR-690 overexpression enhances Runx2-induced osteogenic differentiation of C2C12 cells, whereas knockdown of miR-690 leads to the opposite effect (Fig. 2b–d). These findings suggest that miR-690 is an osteogenesis-related miRNA and positively regulates Runx2-induced osteogenic differentiation.

As shown in Fig. 2a, miR-690 expression was rapidly induced following activation of Runx2, suggesting that the transcriptional activation of *mir*-*69*0 is dependent upon Runx2 expression. It further suggested that *mir*-*69*0 may be a direct target of Runx2. To define the molecular mechanism by which Runx2 regulates the expression of miR-690, we performed in silico analysis and identified four potential Runx2-binding sites in the −1 kb genomic sequence upstream of the *mir*-*690* stem loop (Fig. 3a). EMSA analysis using nuclear extract from Dox-treated C2C12/Runx2^Dox^ cells demonstrated specific binding of Runx2 for the Runx2 motif at position −246 (site2, Runx2-2)(Fig. 3b). However, the data obtained from these artificial in vitro systems may not necessarily reflect the regulation and function of the endogenous gene. To address this concern, we used ChIP analysis to corroborate the EMSA data. This study clearly demonstrated that Runx2 is specifically associated with the putative promoter of *mir*-*690* during osteogenic differentiation of C2C12 cells (Fig. 3c). Together, our findings reveal that *mir*-*690* could be a direct target of Runx2 during osteogeneis.

To study the molecular mechanism by which miR-690 regulates Runx2-induced osteogenic differentiation, we searched for potential target genes that have an established function in inhibiting osteogenesis. Interestingly, the 3′UTR of *p65* possesses two match sites to the miR-690 seed region. p65, a subunit of NF-κB, which can function either as a homodimer or as a classical heterodimer with the p50 subunit of NF-κB [40]. Indeed, *p65* 3′UTR luciferase reporter assays confirmed that *p65* is a direct target of miR-690 (Fig. 4c). We showed that miR-690 mediates translational repression but not mRNA degradation of *p65* (Fig. 4d, e). Overexpression of p65 inhibits Runx2-induced osteogenic differentiation (Fig. 5a), and the effect of p65 on Runx2-induced late osteoblast differentiation is partially mediated through upregulating the NF-κB downstream target IL-6 (Figs. 5, 6). In this study, the target that mediates the effect of p65 on Runx2-induced early osteoblast differentiation was not investigated, and other previously reported (e.g. IL-8, ICAM-1, miR-17, miR-21, miR-27b, and miR-30b) [41–43] or undisclosed targets might participate in this process.

It has been reported that p65 can inhibit BMP-2-induced osteogenic differentiation by decreasing the DNA binding of the Smad complex [21]. Tumor necrosis factor alpha (TNFα), a proinflammatory cytokine, can exert its negative effect on osteoblast differentiation and bone formation through the NF-κB p50/p65 heterodimer [40]. Runx2 stability can be reduced by TNFα through upregulation of E3 ubiquitin ligases Smad ubiquitin regulatory factor 1 [44]. WW domain containing E3 ubiquitin protein ligase 1, which was significantly upregulated in mesenchymal stem cells (MSCs) from TNFα-transgenic mice [45], was found to be recruited by Schnurri-3 and to mediate Runx2 degradation in a ubiquitin–proteasome-dependent manner [46]. MiR-3077-5p, a miRNA upregulated by TNFα in MSCs from osteoporosis bone marrow of mice, was demonstrated to directly target Runx2 and to inhibit Runx2 translation [47]. These findings raise the possibility that Runx2 function could be negatively regulated by the NF-κB p50/p65 heterodimer. Thus, it is necessary to inactivate the canonical NF-κB pathway by Runx2 through miR-690-mediated downregulation of p65 during this differentiation process.

In addition, other undisclosed miR-690 targets may contribute to the phenotypic effects observed on inhibition or overexpression of miR-690. There are more than 100 predicted targets for miR-690 in DIANA-microT, miRDB, and miRWalk [18–20], some of which are very relevant in the process of osteoblast differentiation and bone formation (e.g., Bcl2, Igfbp5, Smad7, Crem, Stat3, and Mmp28). The involvement of other potential osteogenic targets should be elucidated in future studies.

## Conclusions

Taken together, our study provides a comprehensive profiling of Runx2-regulated miRNAs during osteogenic differentiation of C2C12 myogenic progenitor cells. MiR-690, a newly identified Runx2-targeted miRNA, exerts its positive effect on Runx2-induced osteogenic differentiation by inactivating the NF-κB pathway via the downregulation of the NF-κB subunit p65. Our findings enrich the knowledge of the Runx2-centered regulatory network that functions in osteoblast differentiation and bone formation, and may give some tips for treating human osseous defects.

## Methods

### Primers and probes

All primers and probes are available in Additional file 11: Tables S1–S7.

### Cell culture

The establishment of C2C12/Runx2^Dox^ sub-line has been described previously [14]. C2C12/Runx2^Dox^ cells were maintained in Dulbecco’s modified Eagle medium (DMEM) (Invitrogen, Carlsbad, CA, USA) supplemented with 10 % Tet System Approved fetal bovine serum (FBS) (Clontech, Mountain View, CA, USA). Hygromycin B and G418 were purchased from Invitrogen and added into the medium at the concentration of 500 μg/ml and 800 μg/ml, respectively. For osteogenic differentiation, the C2C12/Runx2^Dox^ cells were cultured in medium containing Dox (10 μg/ml) (Clontech) and BMP-2 (100 ng/ml) (R&D Systems, Minneapolis, MN, USA) respectively. For myogenic differentiation, C2C12/Runx2^Dox^ cells were grown in growth medium consisting of high-glucose DMEM supplemented with 10 % FBS (Invitrogen). At 80–90 % confluence, myogenic differentiation was induced by replacing the growth medium with differentiation medium consisting of DMEM supplemented with 2 % horse serum (Invitrogen). Additionally, the establishment and culture of C2C12/Vector^Dox^ cells were similar to that of C2C12/Runx2^Dox^ cells.

### MiRNA microarray analysis

Total RNA containing small RNA was extracted from Dox-treated C2C12/Runx2^Dox^ cells by using a mirVana miRNA Isolation Kit (Ambion, Austin, TX, USA). MiRNA profiling was performed using GeneChip miRNA Array version 1.0 (Affymetrix, Santa Clara, CA, USA). The array comprised 6703 mature microRNA sequences of 71 organisms from the Sanger miRNA database (V.11) and an additional 922 encompassed human snoRNAs and scaRNAs (from Ensembl database and snoRNABase).

Microarray experiments were performed according to the manufacturer’s instructions. Briefly, 1 μg total RNA was labeled with Biotin FlashTag Biotin Labeling Kit (Affymetrix). The labeling reaction was hybridized on the miRNA Array in Affymetrix Hybridization Oven 640 (Affymetrix) at 48 °C for 16 h. The arrays were stained with Fluidics Station 450 using fluidics script FS450_0003 (Affymetrix), and then scanned on a GeneChip microarray scanner (Affymetrix).

### Microarray data analysis

MiRNA probe outliers were defined as per the manufacturer’s instructions (Affymetrix) and further analyzed for data summarization, normalization and quality control by using the miRNA QC Tool software (www.affymetrix.com). To select the differentially expressed miRNAs, we used threshold values of ≥1.5 and ≤−1.5-fold change and a FDR significance level of <5 %. The data was Log2 transformed and median centered by miRNAs using the Adjust Data function of CLUSTER 3.0 software (Michiel de Hoon, Human Genome Center, University of Tokyo, Tokyo, Japan), then further analyzed with hierarchical clustering with average linkage. Finally, we performed tree visualization by using Java Treeview (Stanford University School of Medicine, Stanford, CA, USA).

### Reverse transcription PCR

Total RNA was extracted by Trizol reagent (Invitrogen). A 20 μl reaction mixture containing 1 μg of total RNA was reversely transcribed to cDNA using RT Ace reverse transcriptase (Toyobo, Osaka, Japan). Real-time qPCR was performed on the cDNA to examine the expression levels of mRNAs and miRNAs. The PCR reaction mix was prepared using SYBR Green PCR Master Mix (Takara, Shiga, Japan). PCR conditions were as follows: 95 °C for 30 s, 40 cycles of denaturation at 95 °C for 5 s, annealing at 60 °C for 30 s, and extension at 72 °C for 15 s. PCR was carried out using the Real-Time PCR Detection System Rotor-Gene 6000 (Corbett Research, Mortlake, NSW, Australia). Relative expression levels were calculated as ratios normalized against those of 18S rRNA or U6 snRNA. The data from real-time qPCR were analyzed by the ΔCt method, and the ΔCt value was determined by subtracting the 18S rRNA Ct value from the target gene Ct value or the U6 snRNA Ct value from the target miRNA Ct value. The ΔCt of the treated cells (ΔCts) was subtracted from the ΔCt of the untreated cells (ΔCtu) (ΔΔCt = ΔCts – ΔCtu), and the expression level for a target gene or miRNA in the treated cells compared with the level in the untreated cells was calculated as follows: *x*-fold of untreated control = $2^{{ - {\Delta \Delta }{\text{C}}_{\text{t}} }}$.

### Western blot analysis

Cells were washed with PBS and lysed with mammalian protein extraction reagent from M-PER (Thermo Fisher Scientific, Waltham, MA, USA) supplemented with protease inhibitor mixture tablet (Roche, Indianapolis, IN, USA). Protein concentrations were determined by BCA protein assay (Thermo Fisher Scientific). For western blots, total proteins (30–50 μg) were resolved by 12 % SDS-PAGE gel and transferred to polyvinylidene fluoride membranes (Millipore, Bedford, MA, USA). Membranes were blocked with 5 % non-fat milk for 1 h at room temperature and incubated with primary antibody diluted in 2 % non-fat milk at 4 °C overnight, followed by incubation with the second antibody conjugated to horseradish peroxidase (HRP) for 1 h at room temperature and measured with the enhanced chemiluminescence system (Perkin-Elmer Life and Analytical Sciences, Waltham, MA, USA) and recorded on X-ray films (Fuji, Tokyo, Japan). Protein expression was normalized by β-actin. Primary antibodies used for blotting were anti-p65 (Cell Signaling Technology, Beverly, MA, USA), anti-Flag (Sigma, St Louis, MO, USA), and anti-β-actin (Cell Signaling Technology). Anti-rabbit IgG-HRP (Cell Signaling Technology) were used to detect primary antibodies.

### Immunofluorescence

Cells were washed with PBS, then fixed with 4 % formaldehyde in PBS for 20 min, and finally permeabilized with 0.2 % Triton X-100 solution for 5 min. Cells were incubated with anti-Flag antibody for 1 h, washed three times with PBS, and then incubated with appropriate Alexa Fluor 555-conjugated secondary antibody and washed again three times in PBS. Coverslips were mounted with ProLong Gold antifade reagent with DAPI (Invitrogen), and examined using a Leica fluorescence microscope (Leica, Wetzlar, Germany).

### *In vitro* osteoblastogenesis and cell staining

Cells (C2C12/Runx2^Dox^ and C2C12/Vector^Dox^ cells) were cultured in DMEM containing Dox, β-glycerophosphate disodium (10 mM) and ascorbic acid (50 μg/ml). The medium was changed every 3 days. For ALP staining and Alizarin red staining, the detailed procedures were described previously [48, 49].

### Transfection

To examine the effect of miR-690 on Runx2-induced osteogenic differentiation, the miR-690 mimics and anti-miR-690 oligos purchased from GenePharma (Shanghai, China) were used to promote and inhibit miR-690 activity, respectively. Negative controls (miR and anti-miR control) were used for both reactions. To examine the effect of p65 and IL-6 on Runx2-induced osteogenic differentiation, the cDNAs encoding mouse full-length p65 and IL-6 were obtained by PCR and cloned into the expression vector pcDNA3.1 (Invitrogen), and the IL-6-targeting siRNA oligos (si-IL-6) was purchased from Sigma. Negative controls (pcDNA3.1 and si-NC oligos) were also used for these reactions. For transfection, Lipofectamine 2000 (Invitrogen) was mixed with above-mentioned RNAs (50 nM/100 nM) or vectors (2.5 μg/well) according to the manufacturer’s instructions, and these solutions were directly mixed with C2C12/Runx2^Dox^ cells in 6-well culture plates. For osteogenic differentiation, the medium was replaced with fresh medium containing 10 % FBS and 10 μg/ml of Dox at 4 h after transfection.

### Luciferase reporter assay

A 697-bp fragment of the *p65* 3′UTR containing the predicted binding sites for miR-690 was amplified from mouse genomic DNA using specific primers. Amplicon was cleaved with *Xho*I and *Not*I (MBI Fermentas, Burlington, ON, Canada) and cloned in between the *Xho*I and *Not*I cleavage sites of psiCHECK-2 vector (Promega, Madison, WI, USA) downstream of the *Renilla* luciferase reporter gene. The resulting construct was named psiCHECK-2-*p65*. MiR-690 (50 nM), miR-690-mut (mimics containing a mutated miR-690 seed sequence, 50 nM) (GenePharma), or miR control (50 nM) was co-transfected with psiCHECK-2-*p65* reporter (200 ng) into C2C12/Runx2^Dox^ cells using Lipofectamine 2000. After 48 h, firefly and renilla luciferase activity were measured in cell lysates using a Dual-Luciferase Reporter Assay System (Promega) on a Fusion plate reader (Perkin-Elmer life and Analytical Sciences). Firefly luciferase activity was used for normalization and as an internal control for transfection efficiency.

### EMSA

Nuclear extracts were prepared from Dox-treated C2C12/Runx2^Dox^ cells according to the manufacturer’s instructions (Active Motif, Carlsbad, CA, USA). EMSA was performed using the EMSA kit (Promega). For supershift assays, an antibody against Flag-Runx2 (anti-Flag antibody) (Sigma) or normal mouse IgG (Sigma) was added to the reaction mixture and incubated 25 min before the addition of the labeled oligonucleotide. All DNA–protein complexes were resolved by electrophoresis on 5 % native polyacrylamide gels and transmembrane to immobilon-Ny + (Millipore).

### ChIP assay

ChIP assay was performed according to the manufacturer’s instructions (Active Motif). Briefly, the crosslinked protein-DNA complexes prepared from Dox-treated C2C12/Runx2^Dox^ cells were incubated with 4 μg of the anti-Flag antibody (Sigma) or normal mouse IgG (Sigma) and rotated at 4 °C overnight, and then were incubated with protein G beads at 4 °C for 4 h. The complexes were eluted with buffer containing 1 % SDS and 0.1 M NaHCO_3_, and crosslinks were reversed at 65 °C. DNA was recovered by phenol–chloroform extraction and ethanol precipitation and then subjected to PCR analysis. Amplified products were electrophoresed through 2 % agarose gel and visualized by Goldview staining.

### Statistical analysis

All data were expressed as the mean ± standard deviation (SD) (n = 3), and statistical analysis was performed using SPSS software (version 16.0, Chicago, USA). Differences between two groups were analyzed by the unpaired Student’s *t* test, and differences between multiple groups were analyzed by ANOVA. *P* < 0.05 was considered statistically significant.

## Additional files

10.1186/s13578-016-0073-y C2C12/Runx2^Dox^ cells are actually the wild type C2C12 myogenic progenitor cells in the absence of Dox. (**A**) C2C12/Runx2^Dox^ cells were cultured in medium containing 2 % horse serum for different times. Total RNA prepared from cells at the indicated times was subjected to real-time qPCR analysis. Data are presented as mean ± SD (n = 3). **P* < 0.05 compared with untreated cells (0 d). (**B**) C2C12/Runx2^Dox^ cells were treated with Dox and BMP-2 for 9 days respectively, and the ALP activity was determined by ALP staining. Similar results were obtained in three independent experiments.
10.1186/s13578-016-0073-y. The expression of Flag-Runx2 and *Myog* during Runx2-induced osteogenic differentiation. (**A**) C2C12/Runx2^Dox^ cells were treated with Dox for the times indicated and subjected to western blot analysis with anti-Flag antibody to detect Flag-Runx2. Similar results were obtained in three independent experiments. (**B**) C2C12/Runx2^Dox^ cells were treated with Dox for the times indicated and subjected to real-time qPCR analysis for *Myog* mRNA. Data are presented as mean ± SD (n = 3). **P* < 0.05 compared with untreated cells (0 h).
10.1186/s13578-016-0073-y Detection of Flag-Runx2 by immunofluorescence microscopy in Dox-treated C2C12/Runx2^Dox^ cells at short (**A**) and long (**B**) incubation times, respectively. Immunofluorescence staining for Flag-Runx2 (red) revealed the nuclear distribution (arrow) of Runx2. Nuclei were visualized by DAPI (blue). Scale bar in micrograph represents 20 μm.
10.1186/s13578-016-0073-y Dox itself has no effect on the osteogenic differentiation of C2C12 cells. In Dox-treated C2C12/vector^Dox^ cells, ALP and matrix mineralizing activity were measured by ALP and Alizarin red staining at days 9 and 28, respectively. Similar results were obtained in three independent experiments.
10.1186/s13578-016-0073-y Clustered miRNA expression patterns of 609 miRNAs are shown graphically. Each row represents a different miRNA, and each column displays miRNA expression at each time point (0, 3, 6, 9, 12, 24, 48, and 72 h). Data values displayed as yellow and blue represent elevated and reduced expression, respectively.
10.1186/s13578-016-0073-y Dox itself has no effect on the expression of miR-690. Real-time qPCR results of miR-690 in Dox-treated C2C12/vector^Dox^ cells. Data are presented as mean ± SD (n = 3).
10.1186/s13578-016-0073-y Effect of miR-690 inhibitor on the mRNA and protein levels of p65. The anti-miR control (100 nM) or anti-miR-690 (100 nM) was transfected into C2C12/Runx2^Dox^ cells, and then the mRNA (**A**) and protein (**B**) levels of p65 were examined by real-time qPCR and western blot respectively. The mRNA level of *p65* was normalized to 18S rRNA. Beta-actin expression was used as a loading control. Similar results were obtained in three independent experiments.
10.1186/s13578-016-0073-y The expression of p65 is decreased at the protein but not the mRNA level during Runx2-induced osteogenic differentiation. C2C12/Runx2^Dox^ cells were treated with Dox for the times indicated and subjected to real-time qPCR analysis for *p65* mRNA (**A**) and western blot analysis with anti-p65 antibody to detect p65 (**B**). Similar results were obtained in three independent experiments.
10.1186/s13578-016-0073-y *IL*-*6* is upregulated by overexpression of p65 in C2C12 cells. The empty vector (pcDNA3.1) or p65 expression vector (pcDNA3.1-*p65*) was transfected into C2C12/Runx2^Dox^ cells. The transfected cells were cultured for 3 days in the absence of Dox, and then subjected to real-time qPCR analysis for *IL*-*6* mRNA (left panel). Data are presented as mean ± SD (n = 3). **P* < 0.05 compared with pcDNA3.1-transfected cells. Western blot was performed to assess the overexpression level of p65 (right panel).
10.1186/s13578-016-0073-y p65 exerts its negative effect on the expression of *OC* partially through IL-6 upregulation. (**A**) C2C12/Runx2^Dox^ cells were transfected with si-NC (50 nM) and si-IL-6 (50 nM), respectively, and then cultured for 3 days in the absence of Dox. The mRNA level of *IL*-*6* was examined by real-time qPCR. **P* < 0.05 compared with si-NC sample. (**B**) si-NC (50 nM) or si-IL-6 (50 nM) was co-transfected with p65 expression vector (pcDNA3.1-*p65*) into C2C12/Runx2^Dox^ cells, and then the transfected cells were treated with Dox for 3 days. The mRNA level of *Alp* and *OC* was examined by real-time qPCR. **P* < 0.05 compared with si-NC sample.
10.1186/s13578-016-0073-y Nucleotide sequence of 3′UTR primer for *p65* WT reporter plasmid. **Table S2.** Nucleotide sequence of primers used for RT-PCR. **Table S3.** Nucleotide sequence of miRNA RT primers. **Table S4.** Nucleotide sequence of primers used for miRNA detection. **Table S5.** Nucleotide sequence of primers used for real-time qPCR analysis. **Table S6.** Nucleotide sequence of the oligonucleotides (probes) used in EMSA. **Table S7.** Nucleotide sequence of primers used for ChIP assay.

## Abbreviations

Runx2: runt-related transcription factor 2
miRNAs: microRNAs
NF-κB: nuclear factor kappa B
OC: osteocalcin
3′UTR: 3′ untranslated region
BMP-2: bone morphogenetic protein 2
IL-6: interleukin-6
ALP: alkaline phosphatase
Dox: doxycycline
qPCR: quantitative PCR
EMSA: electrophoretic mobility shift assay
ChIP: chromatin immunoprecipitation
MDSCs: myeloid-derived suppressor cells
TNFα: tumor necrosis factor alpha
MSCs: mesenchymal stem cells
DMEM: Dulbecco’s modified Eagle medium
FBS: fetal bovine serum
HRP: horseradish peroxidase
SD: standard deviation
