# Supplementary figures and images for: MiR-690, a Runx2-targeted miRNA, regulates osteogenic differentiation of C2C12 myogenic progenitor cells by targeting NF-kappaB p65
Source: Cell Biosci. 2016 Feb 12;6:10. doi: 10.1186/s13578-016-0073-y (PMC4751671; doi:10.1186/s13578-016-0073-y)

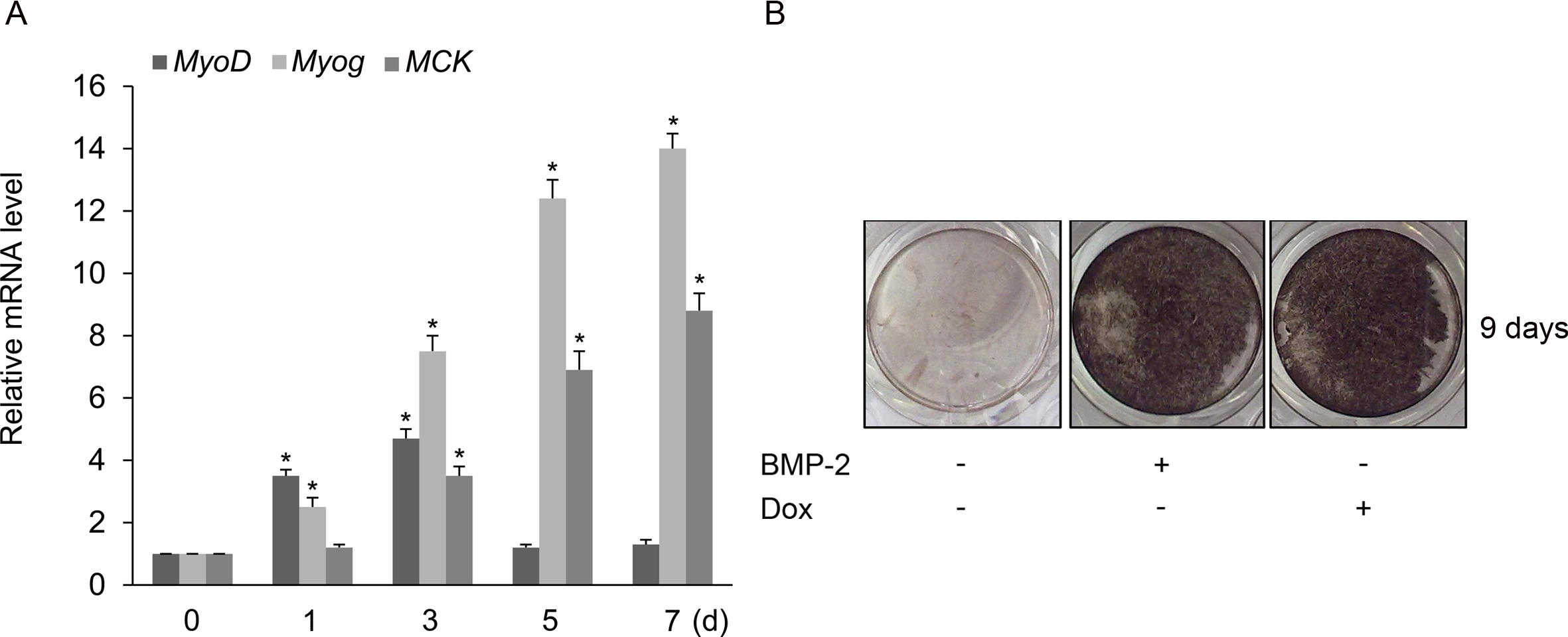

Supplement: Supplementary file 1 — 10.1186/s13578-016-0073-y C2C12/Runx2Dox cells are actually the wild type C2C12 myogenic progenitor cells in the absence of Dox. (A) C2C12/Runx2Dox cells were cultured in medium containing 2 % horse serum for different times. Total RNA prepared from cells at the indicated times was subjected to real-time qPCR analysis. Data are presented as mean ± SD (n = 3). *P < 0.05 compared with untreated cells (0 d). (B) C2C12/Runx2Dox cells were treated with Dox and BMP-2 for 9 days respectively, and the ALP activity was determined by ALP staining. Similar results were obtained in three independent experiments. [file 13578_2016_73_MOESM1_ESM.tif]

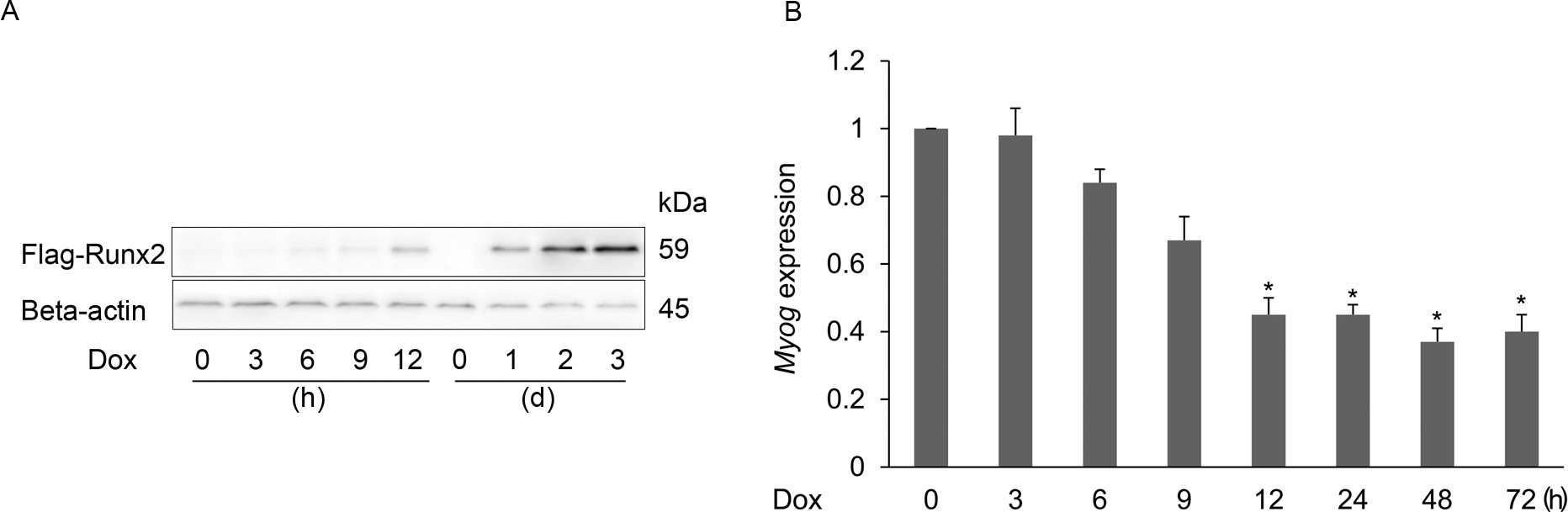

Supplement: Supplementary file 2 — 10.1186/s13578-016-0073-y. The expression of Flag-Runx2 and Myog during Runx2-induced osteogenic differentiation. (A) C2C12/Runx2Dox cells were treated with Dox for the times indicated and subjected to western blot analysis with anti-Flag antibody to detect Flag-Runx2. Similar results were obtained in three independent experiments. (B) C2C12/Runx2Dox cells were treated with Dox for the times indicated and subjected to real-time qPCR analysis for Myog mRNA. Data are presented as mean ± SD (n = 3). *P < 0.05 compared with untreated cells (0 h). [file 13578_2016_73_MOESM2_ESM.tif]

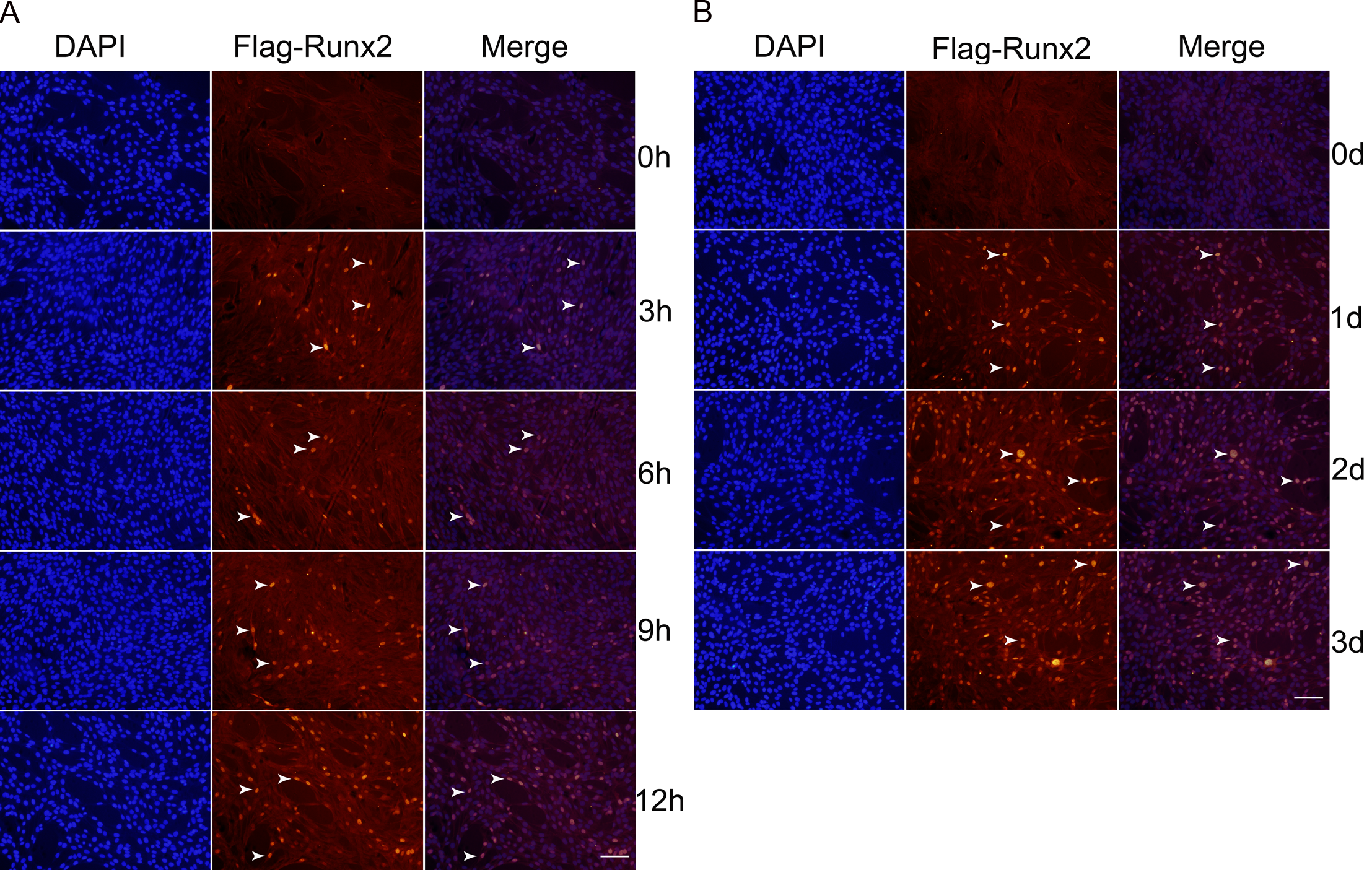

Supplement: Supplementary file 3 — 10.1186/s13578-016-0073-y Detection of Flag-Runx2 by immunofluorescence microscopy in Dox-treated C2C12/Runx2Dox cells at short (A) and long (B) incubation times, respectively. Immunofluorescence staining for Flag-Runx2 (red) revealed the nuclear distribution (arrow) of Runx2. Nuclei were visualized by DAPI (blue). Scale bar in micrograph represents 20 μm. [file 13578_2016_73_MOESM3_ESM.tif]

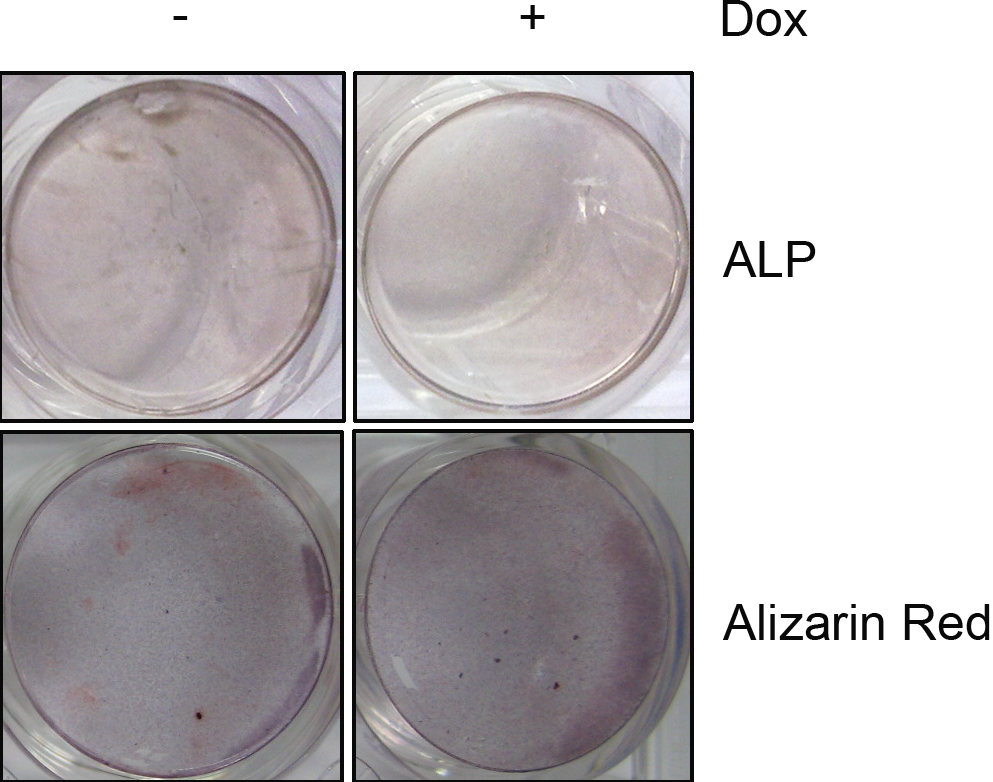

Supplement: Supplementary file 4 — 10.1186/s13578-016-0073-y Dox itself has no effect on the osteogenic differentiation of C2C12 cells. In Dox-treated C2C12/vectorDox cells, ALP and matrix mineralizing activity were measured by ALP and Alizarin red staining at days 9 and 28, respectively. Similar results were obtained in three independent experiments. [file 13578_2016_73_MOESM4_ESM.tif]

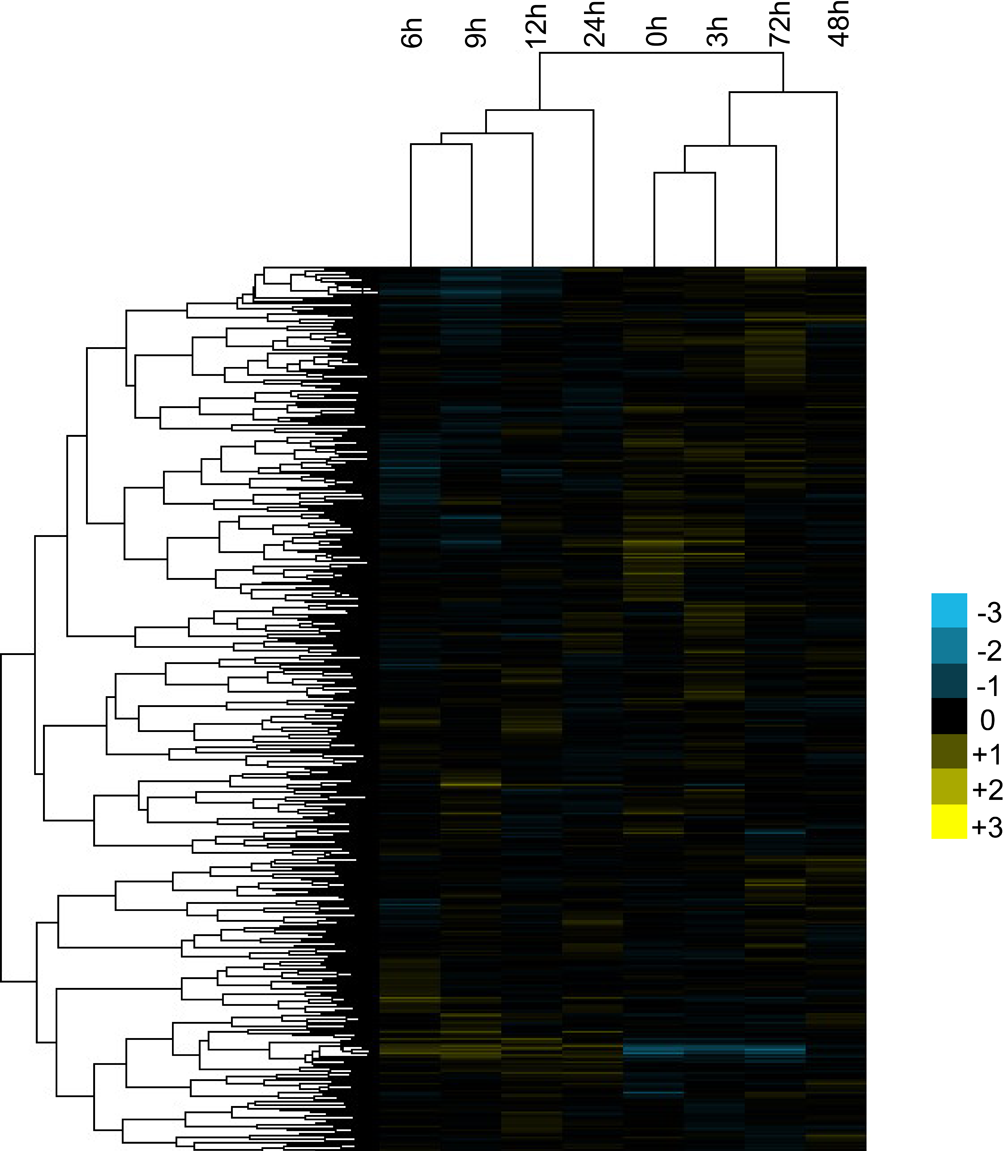

Supplement: Supplementary file 5 — 10.1186/s13578-016-0073-y Clustered miRNA expression patterns of 609 miRNAs are shown graphically. Each row represents a different miRNA, and each column displays miRNA expression at each time point (0, 3, 6, 9, 12, 24, 48, and 72 h). Data values displayed as yellow and blue represent elevated and reduced expression, respectively. [file 13578_2016_73_MOESM5_ESM.tif]

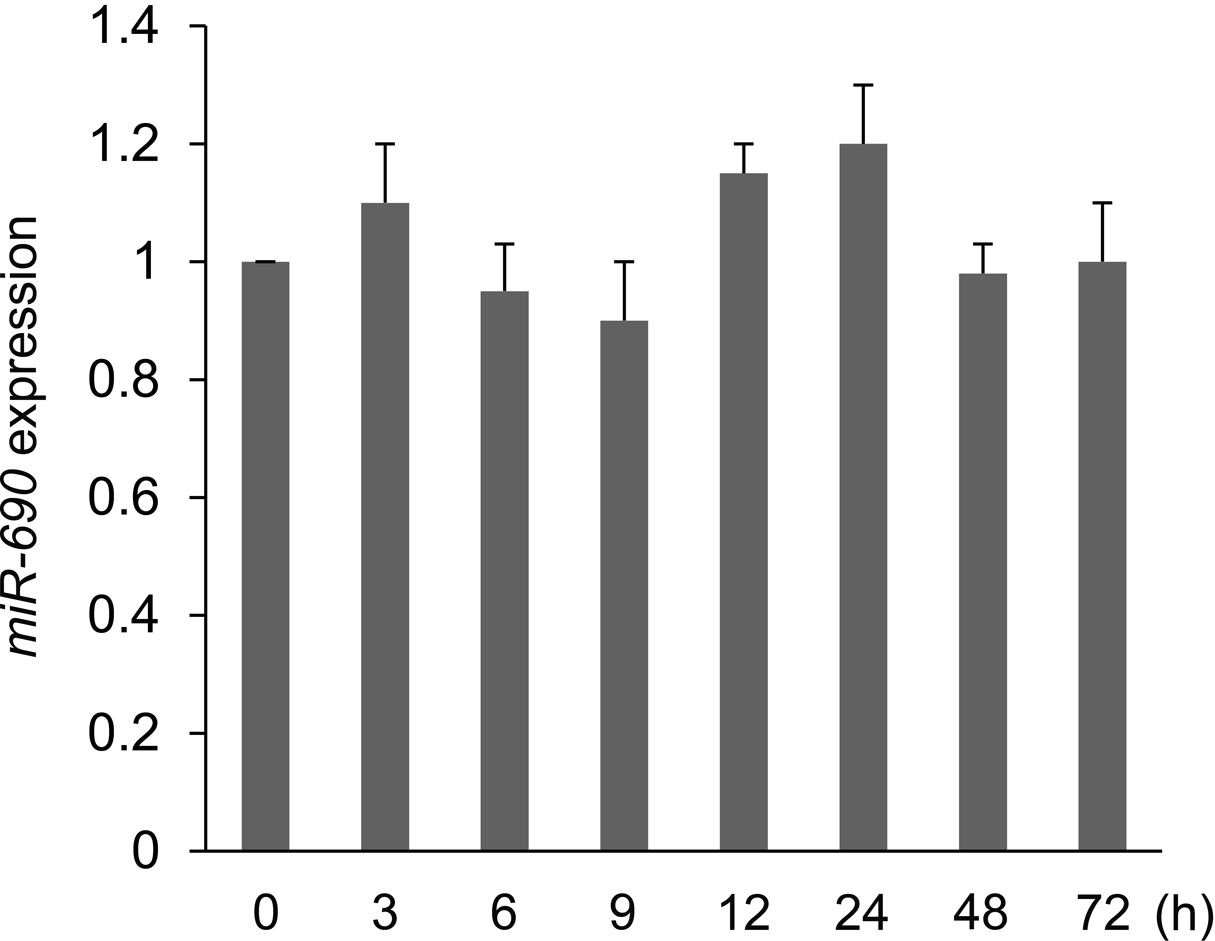

Supplement: Supplementary file 6 — 10.1186/s13578-016-0073-y Dox itself has no effect on the expression of miR-690. Real-time qPCR results of miR-690 in Dox-treated C2C12/vectorDox cells. Data are presented as mean ± SD (n = 3). [file 13578_2016_73_MOESM6_ESM.tif]

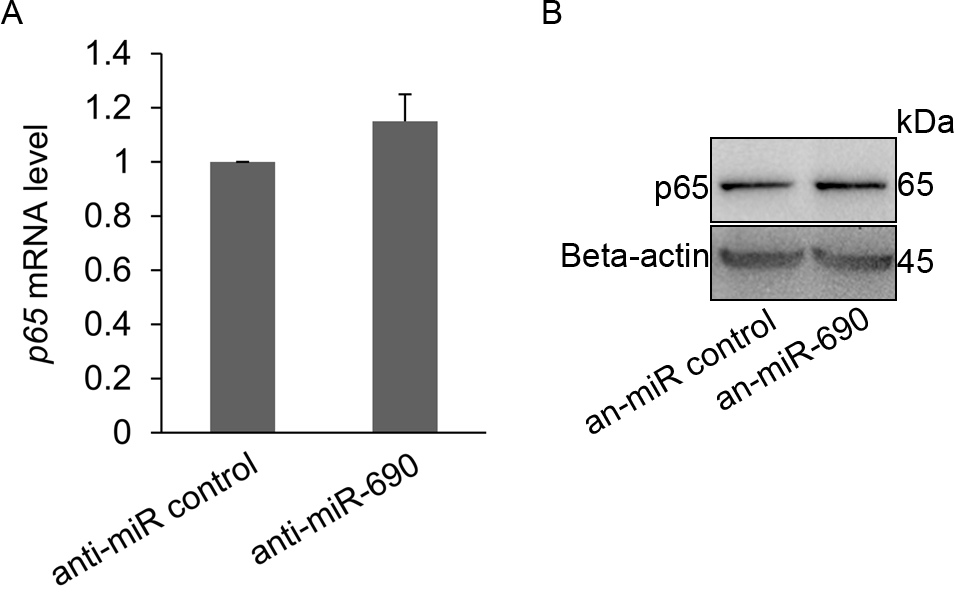

Supplement: Supplementary file 7 — 10.1186/s13578-016-0073-y Effect of miR-690 inhibitor on the mRNA and protein levels of p65. The anti-miR control (100 nM) or anti-miR-690 (100 nM) was transfected into C2C12/Runx2Dox cells, and then the mRNA (A) and protein (B) levels of p65 were examined by real-time qPCR and western blot respectively. The mRNA level of p65 was normalized to 18S rRNA. Beta-actin expression was used as a loading control. Similar results were obtained in three independent experiments. [file 13578_2016_73_MOESM7_ESM.tif]

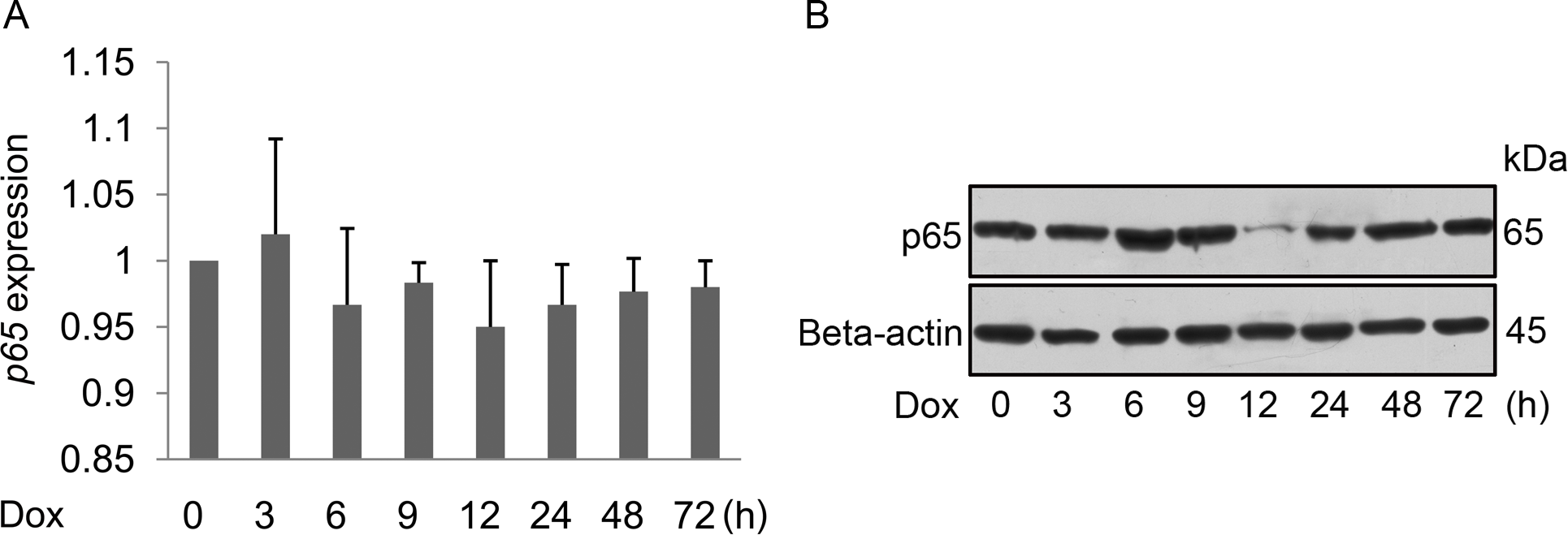

Supplement: Supplementary file 8 — 10.1186/s13578-016-0073-y The expression of p65 is decreased at the protein but not the mRNA level during Runx2-induced osteogenic differentiation. C2C12/Runx2Dox cells were treated with Dox for the times indicated and subjected to real-time qPCR analysis for p65 mRNA (A) and western blot analysis with anti-p65 antibody to detect p65 (B). Similar results were obtained in three independent experiments. [file 13578_2016_73_MOESM8_ESM.tif]

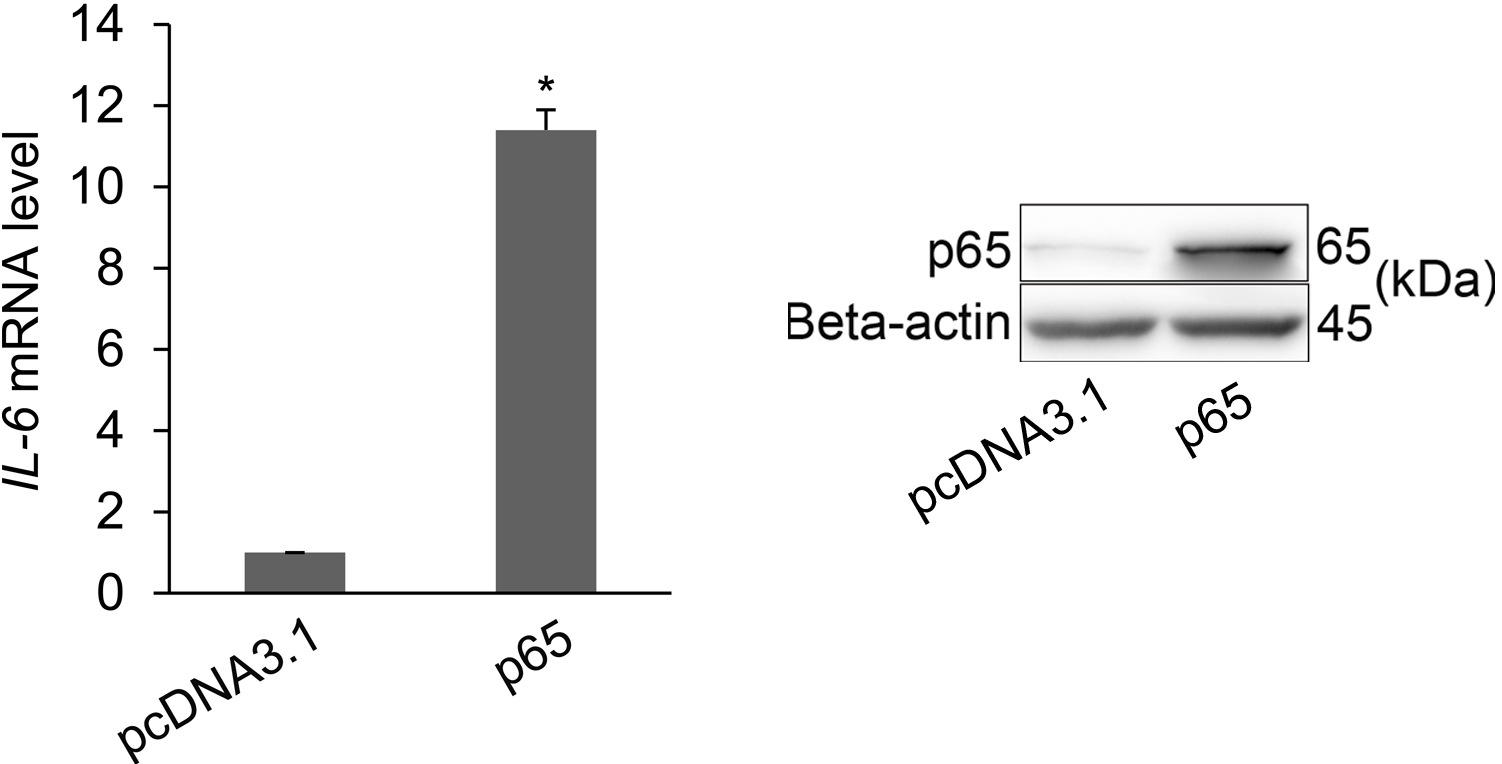

Supplement: Supplementary file 9 — 10.1186/s13578-016-0073-y IL-6 is upregulated by overexpression of p65 in C2C12 cells. The empty vector (pcDNA3.1) or p65 expression vector (pcDNA3.1-p65) was transfected into C2C12/Runx2Dox cells. The transfected cells were cultured for 3 days in the absence of Dox, and then subjected to real-time qPCR analysis for IL-6 mRNA (left panel). Data are presented as mean ± SD (n = 3). *P < 0.05 compared with pcDNA3.1-transfected cells. Western blot was performed to assess the overexpression level of p65 (right panel). [file 13578_2016_73_MOESM9_ESM.tif]

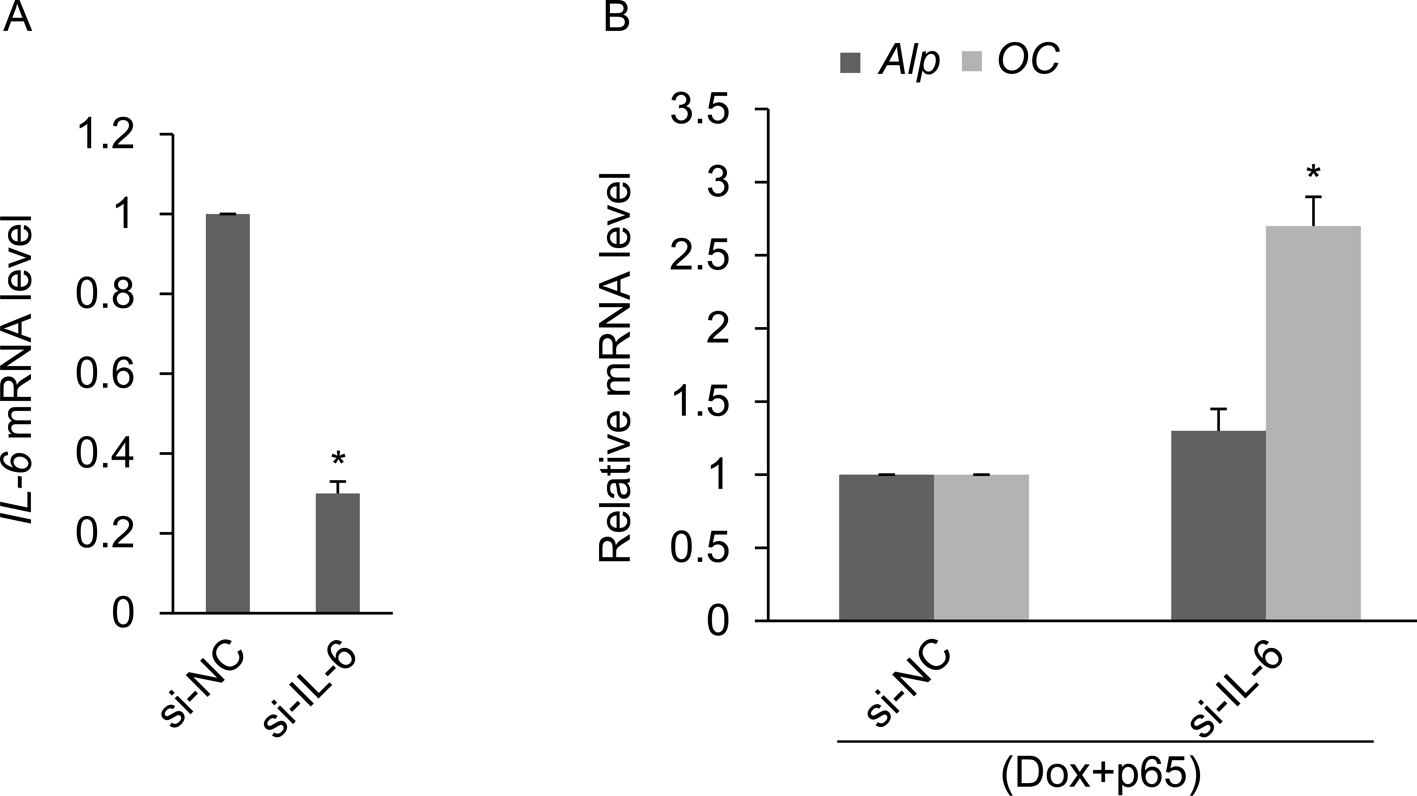

Supplement: Supplementary file 10 — 10.1186/s13578-016-0073-y p65 exerts its negative effect on the expression of OC partially through IL-6 upregulation. (A) C2C12/Runx2Dox cells were transfected with si-NC (50 nM) and si-IL-6 (50 nM), respectively, and then cultured for 3 days in the absence of Dox. The mRNA level of IL-6 was examined by real-time qPCR. *P < 0.05 compared with si-NC sample. (B) si-NC (50 nM) or si-IL-6 (50 nM) was co-transfected with p65 expression vector (pcDNA3.1-p65) into C2C12/Runx2Dox cells, and then the transfected cells were treated with Dox for 3 days. The mRNA level of Alp and OC was examined by real-time qPCR. *P < 0.05 compared with si-NC sample. [file 13578_2016_73_MOESM10_ESM.tif]
