# Supplementary material for: MiR-690, a Runx2-targeted miRNA, regulates osteogenic differentiation of C2C12 myogenic progenitor cells by targeting NF-kappaB p65
Source: Cell Biosci. 2016 Feb 12;6:10. doi: 10.1186/s13578-016-0073-y (PMC4751671; doi:10.1186/s13578-016-0073-y)
Supplement: Supplementary file 11 — 10.1186/s13578-016-0073-y Nucleotide sequence of 3′UTR primer for p65 WT reporter plasmid. Table S2. Nucleotide sequence of primers used for RT-PCR. Table S3. Nucleotide sequence of miRNA RT primers. Table S4. Nucleotide sequence of primers used for miRNA detection. Table S5. Nucleotide sequence of primers used for real-time qPCR analysis. Table S6. Nucleotide sequence of the oligonucleotides (probes) used in EMSA. Table S7. Nucleotide sequence of primers used for ChIP assay. [file 13578_2016_73_MOESM11_ESM.pdf]

Table S1 Nucleotide sequence of 3'UTR primer for *p65* WT reporter plasmid

**Name**	**Sequence**
p65 sence	5'-TGCTCAGAGCACCAGGTTTCAG-3'
p65 anti-sence	5'-CAGCGTGATAAGACATTTATTAGTTC-3'

Table S2 Nucleotide sequence of primers used for RT-PCR

Gene	Sequence
p65	5’-ATGGACGATCTGTTTCCCCTCATC-3’ (Forward)<br>5’-TTAGGAGCTGATCTGACTCAAAAGAG-3’(Reverse)
IL-6	5’-ATGAAGTTCCTCTCTGCAAGAG-3’ (Forward)<br>5’-CTAGGTTTGCCGAGTAGATCTC-3’ (Reverse)

Table S3 Nucleotide sequence of miRNA RT primers

miRNA	Sequence
miR-690	5’-CTCAACTGGTGTCGTGGAGTCGGCAA TTCAGTTGAGTTTGGTT-3’
U6	5’-AACGCTTCACGAATTTGCGT-3’

Table S4 Nucleotide sequence of primers used for miRNA detection

Name	Sequence
miR-690 Forward	5’-ACACTCCAGCTGGGAAAGGCTAGGCTCACAAC-3’
miR-690 Reverse (Universal Reverse)	5’-CTCAACTGGTGTCGTGGA-3’
U6 Forward	5’-CTCGCTTCGGCAGCACA-3’
U6 Reverse	5’-AACGCTTCACGAATTTGCGT-3’

Table S5 Nucleotide sequence of primers used for real-time qPCR analysis

Gene	Sequence
Runx2	5’-TAAGAAGAGCCAGGCAGGTGC-3’(Forward)<br>5’-AGGTACGTGTGGTAGTGAGTG-3’(Reverse)
Alp	5’-GGGTGGACTACCTCTTAGGTC-3’(Forward)<br>5’-ATGATGTCCGTGGTCAATCCTG-3’(Reverse)
OC	5’-GCTACCTTGGAGCCTCAGTC-3’(Forward)<br>5’-GATCAAGTCCCGGAGAGCAG-3’(Reverse)
p65	5’-CAGCACCATCAACTTTGATGAG-3’ (Forward)<br>5’-GAGGTACCATGGCTGAGGAAG-3’(Reverse)
IL-6	5’-CCAGAGTCCTTCAGAGAGATAC-3’(Forward)<br>5’-GCCGAGTAGATCTCAAAGTGAC-3’(Reverse)
Myog	5’-ACTGAGATTGTCTGTCAGGCTG-3’(Forward)<br>5’-GGGTGTTAGCCTTATGTGAATG-3’(Reverse)
MyoD	5’-TGATGGCATGATGGATTACAGC-3’(Forward)<br>5’-GTGGAGATGCGCTCCACTATG-3’(Reverse)
MCK	5’-TGATCTCCATGGAGAAGGGAG-3’(Forward)<br>5’-AGGTGCTCGTTCCACATGAAG-3’(Reverse)
18S RNA	5’-AATTGACGGAAGGGCACCAC-3’(Forward)<br>5’-CACCAACTAAGAACGGCCATG-3’(Reverse)

Table S6 Nucleotide sequence of the oligonucleotides (probes) used in EMSA

Name	Sequence
miR-690-Runx2-2-WT	5'-AGGGTTGCGAACCACTGAGCTAGAGT-3'
miR-690-Runx2-2-Mut	5'-AGGGTTGCG*GGCCTTCCC*GCTAGAGT-3'

The underlined sequences represent the putative Runx2-2 site. The italic letters indicate the mutated Runx2-2 site.

Table S7 Nucleotide sequence of primers used for ChIP assay

Name	Sequence
Primer-1(Runx2-2)	5’-CACTCATAACTCGTCTCCTC-3’(Forward)<br>5’-GCCTGAGAGCTTAACTCTAG-3’(Reverse)
Primer-2(unrelated region)	5’-GTGTTCCCAGCCTGTTAGAGA-3’(Forward)<br>5’-GAGACAGAGACAGAGTCTTTC-3’(Reverse)
